# Physics-Based Modeling of Sparse Single-Cell Hi-C Uncovers Structural and Epigenetic Variability

**DOI:** 10.3390/ijms27114803

**Published:** 2026-05-26

**Authors:** Francesca Vercellone, Sumanta Kundu, Andrea Esposito, Andrea M. Chiariello, Mattia Conte, Alex Abraham, Andrea Fontana, Florinda Di Pierno, Sougata Guha, Ciro Di Carluccio, Matteo Olimpo, Mario Nicodemi, Francesco Paolo Casale, Simona Bianco

**Affiliations:** 1 Dipartimento di Ingegneria Elettrica e delle Tecnologie dell’Informazione—DIETI, Università di Napoli Federico II, Via Claudio 21, 80125 Naples, Italy; francesca.vercellone@unina.it; 2 Istituto Nazionale di Fisica Nucleare, Sezione di Napoli, Complesso Universitario di Monte Sant’Angelo, Ed. 6, Via Cintia, 80126 Naples, Italy; sumanta.kundu@na.infn.it (S.K.); andresposito@na.infn.it (A.E.); chiariello@na.infn.it (A.M.C.); mattia.conte@na.infn.it (M.C.); alexabraham492@gmail.com (A.A.); andrea.fontana@unina.it (A.F.); florinda.dipierno@unina.it (F.D.P.); guha@na.infn.it (S.G.); ciro.dicarluccio@na.infn.it (C.D.C.); matteo.olimpo@unina.it (M.O.); mario.nicodemi@na.infn.it (M.N.); 3 Dipartimento di Fisica, Università di Napoli Federico II, Complesso Universitario di Monte Sant’Angelo, 80126 Naples, Italy; 4 Dipartimento di Ingegneria Chimica dei Materiali e Della Produzione Industriale—DICMaPI, Università degli Studi di Napoli Federico II, Piazzale Vincenzo Tecchio 80, 80125 Naples, Italy; 5 Institute of AI for Health, Helmholtz Zentrum München—German Research Center for Environmental Health, 85764 Neuherberg, Germany; francescopaolo.casale@helmholtz-munich.de; 6 Helmholtz Pioneer Campus, Helmholtz Zentrum München—German Research Center for Environmental Health, 85764 Neuherberg, Germany; 7 School of Computation, Information and Technology, Technical University of Munich, 85748 Garching, Germany

**Keywords:** chromatin architecture, single cell, polymer physics, computational modeling

## Abstract

Chromatin conformation capture technologies have revealed the complex 3D organization of the genome and its key regulatory role. Single-cell Hi-C (scHi-C) maps this architecture at single-cell level, but its sparse nature makes data interpretation challenging, and tools for their analysis remain limited. Here, we present a physics-based framework that combines polymer modeling with computational methods to reconstruct full 3D genome structures from sparse scHi-C data. Using both artificial and experimental data, we show that our approach imputes missing contacts and recovers accurate structures validated against independent Hi-C and established polymer models. Applied to scHi-C from a 15 Mb region of human HeLa-S3 cells as a case study, the method uncovers distinct structural classes defined by the spatial distribution of chromatin binding domains. The reconstructed models enable robust downstream analyses, including the identification of single-cell topologically associated domains (TADs), which appear highly variable across cells yet tend to accumulate around those observed in bulk. Importantly, the inferred 3D polymer models capture diverse epigenetic signatures, with active chromatin domains exhibiting greater structural variability than repressive ones across single cells. Overall, our study provides a mechanistic and interpretable framework to analyze sparse scHi-C data, highlighting how polymer physics can be leveraged to uncover genome architecture and its functional variability at single-cell resolution.

## 1. Introduction

Chromatin has a complex 3D organization in the cell nucleus, as uncovered through several experimental techniques [1], notably chromosome conformation capture (3C)-based technologies such as Hi-C [2]. Hi-C can be used to generate genome-wide pairwise contact maps, providing the contact frequency between any pair of loci along the genome. Such technologies revealed the functional patterns of 3D genome organization across length scales including A/B compartments, topologically associating domains (TADs), and loops [2,3,4]. Importantly, such organization plays a central role in regulating key biological functions, such as gene transcription, and its variability has been linked, e.g., to cell type, differentiation and developmental stage, while disruptions have been associated with diseases [5].

To deconvolve the complexity of Hi-C contact maps data and reconstruct the details of genome 3D structures from observed contacts between loci, a variety of physics-based models have been developed. A class of models, exemplified by the Strings and Binders Switch (SBS) model [6,7], describes a classical scenario in which specific binding sites along the chromatin fiber interact with cognate diffusing molecules, such as transcription factors, leading to the formation of spatial domains of homologous sites through microphase separation [7,8,9,10,11,12,13,14]. Another major class of models describes loop formation as an active extrusion process driven by DNA-binding proteins such as cohesin, constrained by boundary elements such as CTCF binding sites [15,16,17]. Complementarily, data-driven approaches have been developed to infer the 3D chromatin architecture directly from sequence and epigenomic information, independently from the underlying physical mechanism [18,19,20,21,22,23,24,25,26].

While bulk Hi-C studies have led to fundamental insights, they inherently average contact data over large populations of cells, obscuring cell-to-cell heterogeneity that may be functionally meaningful. Emerging single-cell Hi-C (scHi-C) technologies now provide the means to dissect genome architecture at single-cell level, revealing variability in chromatin conformation that correlates with cell type and epigenetic state [27,28,29,30,31,32]. In this context, bulk Hi-C maps can be regarded as ensemble averaged genome conformations, whereas single-cell data capture individual conformational realizations. However, the typical sparsity and noise of scHi-C data pose major challenges to theoretical models for 3D structure reconstruction [33].

To address this, several data-driven approaches have been proposed to infer genome organization from scHi-C data. Graph-based methods, such as scHiCluster [34], use convolutional smoothing and random walk with restart algorithms to impute missing contacts in the sparse single-cell matrices. Bayesian probabilistic models like SIMBA3D [35] and Si-C [36] incorporate data likelihood and folding constraints to estimate posterior distributions over possible genome structures. Deep learning methods, exemplified by Higashi [33,37], employ hypergraph representation learning to capture low-dimensional representations of chromatin contacts across cells, enabling the imputation of contact maps followed by the inference of spatial organization features. More recently, Tensor-FLAMINGO [38] introduced a low-rank tensor completion model that leverages global, multi-cell structural dependences, enhancing denoising and enabling higher-resolution reconstruction compared to local-context models. On the other hand, biophysical approaches have been developed for reconstructing 3D genome structures consistent with experimental data. For example, the recent Hi-BDiSCO [39] employs Brownian dynamics simulations at the mesoscale to generate an ensemble of 3D structures consistent with both experimental contact data and underlying physical laws. However, these approaches generally require dense contact maps to constrain physical simulations, precluding their direct use on available sparse scHi-C datasets.

Thus, while data-driven methods excel at statistical imputation and physics-based approaches provide mechanistic insight, there is a lack of approaches that directly integrate biophysical modeling with sparse scHi-C data to reconstruct complete and interpretable 3D genome structures grounded in physical principles.

Here we introduce a framework that integrates polymer physics with computational approaches to both recover missing contacts and reconstruct full, physically grounded 3D genome structures from sparse scHi-C data.

To model chromatin folding, we use the SBS polymer model [7,11]. The model binding site profiles for a given genomic region are obtained by the PRISMR optimization method [40] and have been shown to well match known epigenetic states [13]. This consolidated polymer physics framework has been successfully applied to derive 3D structures from bulk Hi-C, GAM and SPRITE data across chromosomes, cell types and organisms, as validated by numerous independent experiments. However, its performance on sparse contact data such as single-cell Hi-C still needs to be tested.

Here we show that this framework can be reliably extended to model sparse scHi-C data, establishing its feasibility, interpretability, and robustness through a proof-of-principle case study. The approach allows us to obtain accurate 3D reconstructions of the genome architecture and information on the position of DNA binding sites driving chromatin folding at the single-cell level. The obtained models also permit us to improve single-cell contact maps with missing contacts imputed from physics, and more generally provide the base for more robust downstream analyses such as the identification of chromatin folding features, e.g., TADs, in single cells.

First, to provide a controlled validation of our approach, we apply it to artificial sparse data generated by subsampling bulk Hi-C matrices, and demonstrate that it can recover the original, ground truth, Hi-C maps. Next, we employ our method to derive 3D structures from real scHi-C data [28], focusing on a 15 Mb region of human HeLa-S3 cells as a case study, and show that the predicted structures can be classified in different classes based on their binding sites patterns. We validate the inferred structures by comparing their predictions about bulk contacts against independent Hi-C and against independent polymer models based on bulk Hi-C. Additionally, we show that the inferred structures allow us to implement a downstream analysis of contact patterns, notably TAD calling in single cells. Finally, we characterize the biological nature of the predicted single-cell polymer models by comparison with independent epigenetic data, finding that the single-cell model binding domains can be clustered in specific epigenetic classes, having diverse variability, with the domains falling in active classes generally showing higher cell-to-cell variability compared to repressive ones.

In summary, our physics-based computational approach enables the reconstruction of reliable 3D structures from very sparse single-cell contact data, while providing insights into the folding mechanisms and giving us the possibility of robust downstream analysis, overall offering the potential to deepen our comprehension of the dynamic 3D genome organization and its functional role at the single-cell level.

## 2. Results

### 2.1. Polymer Physics-Based Inference of Genome Architecture from Sparse Hi-C Data

To model the 3D organization of genomic regions, we employ the SBS polymer physics model of chromatin folding [7,11]. In this model, a chromatin filament is described as a polymer chain of beads, where specific binding sites distributed along the polymer interact with diffusing cognate molecular binders, driving the folding of the chain into 3D conformations through microphase separation of the different binding site types. The SBS model of a genomic region of interest, i.e., the number and location of the different types of binding sites along the polymer, is obtained by the PRISMR optimization procedure [40]. PRISMR infers from the input contact data only, such as Hi-C, of a genomic region, the minimal set of polymer binding sites that explains the formation of the region contact patterns through the physics mechanism envisaged by the SBS model. Importantly, PRISMR does not require any prior knowledge of ChIP-seq data or known binding proteins. This framework has been successfully applied to infer chromatin architecture from bulk Hi-C, GAM and SPRITE data across multiple organisms, cell types, and genomic scales [7,11,12,13,40,41,42,43], but has not yet been tested on sparse data. Here, we develop a computational procedure (Section 4) based on the PRISMR approach to infer chromatin 3D architecture from sparse single-cell Hi-C.

**Figure 1:**
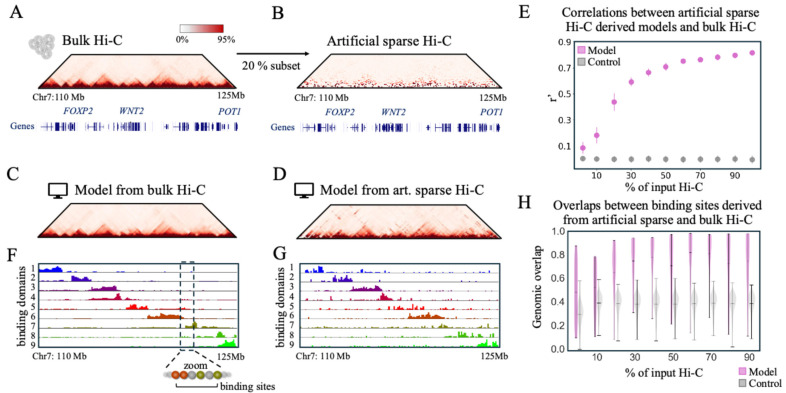
Polymer physics can reconstruct 3D structure from sparse contact data. (**A**) Bulk Hi-C [44] contact matrix of a 15 Mb-wide locus (chr7: 110–125 Mb) at 100 kb resolution in HeLa-S3 cells. Gene annotations from UCSC Genome Browser are shown below. (**B**) Artificial sparse Hi-C matrix obtained by randomly downsampling the bulk Hi-C matrix to 20% of its original entries. The downsampled matrix largely loses the original patterns. (**C**) Contact matrix of the polymer model inferred from bulk Hi-C of the locus has a distance-corrected Pearson correlation with the bulk Hi-C matrix of r′ = 0.82. (**D**) Contact matrix of the polymer model inferred from the artificial sparse Hi-C matrix in (**B**). Despite the input sparsity, the model largely retrieves missing patterns, as also highlighted by its distance-corrected Pearson correlation with the bulk Hi-C matrix (**A**), r′ = 0.51. (**E**) Distance-corrected Pearson correlation, r′, between the original bulk Hi-C matrix and model contact matrices inferred from downsampled Hi-C data at different input percentages (magenta). Each point represents the average over 20 independent, random downsamplings; error bars denote standard deviation. Gray points represent a random control obtained by shuffling contact values within each diagonal of the inferred model contact matrices. (**F**) Genomic location and abundance of the different types of binding sites (binding domains) of the polymer model inferred from bulk Hi-C. Each of the 9 inferred binding domains is visually represented by a different color. (**G**) Distribution of the polymer model binding domains inferred from the artificial sparse Hi-C in (**B**). (**H**) Distribution of overlaps (magenta) between model binding domains found from artificial sparse Hi-C at different downsampling percentages and model binding domains found from bulk Hi-C. Statistical significance was assessed against a random control (gray) obtained by bootstrapping the model binding site positions, using one-sided Mann–Whitney U tests with Benjamini–Hochberg correction for multiple comparisons. Effect sizes, reported as median differences and Cliff’s δ, are provided in Supplementary Table S1.

To validate the robustness of the PRISMR method to sparsity, we first evaluated its performance on artificial sparse data, generated by downsampling ground truth bulk data. To this aim, we used bulk Hi-C data from human HeLa-S3 cells [44] at 100 kb resolution, focusing, as a case study, on a 15 Mb locus on chromosome 7 (hg38, chr7: 110,000,000–125,000,000 bp, Figure 1A). The selected region exhibits the typical structural complexity of Hi-C matrices, comprising an intricate landscape of genes and regulatory features and including typical structural features such as several TADs.

We generated downsampled versions of the bulk Hi-C matrix by randomly retaining a fraction of the original matrix entries and setting the remaining entries to zero (Figure 1B and Figure S1 and Section 4). Next, by using the PRISMR optimization procedure (Section 4), we built polymer models from both original and downsampled Hi-C data (Figure 1C,D). Despite reduced input information, the algorithm produced accurate reconstructions even at low input levels, with the main TAD structures of the locus recovered (Figure 1D and Figure S1) even if they are not visible in the sparse Hi-C data given in input. For example, with just 20% of the data (Figure 1B), we still achieved a distance corrected correlation (Section 4) of r′ = 0.51 between the predicted contact matrix and the original (non-downsampled) bulk Hi-C (Figure 1D).

A systematic evaluation of the model performance showed that r’ increases with the fraction of Hi-C contacts given in input, as expected; however, correlation remains significant at all downsampling levels, providing quantitative validation of the method under increasing sparsity (Figure 1E and Figure S1 and Section 4). Significance was assessed against a control case in which the genomic organization of the inferred contact matrices was randomized by shuffling values within each diagonal (thus preserving genomic distance effects). These randomized matrices showed negligible correlation with the original bulk Hi-C across all input levels, indicating that the method captures specific structural features (Figure 1E, Section 4).

Moreover, we validated the polymer binding site arrangements inferred from these sparse data by checking their similarity with those inferred from the ground truth bulk Hi-C (Figure 1F–H and Figure S1). To compare the binding domains profiles of each sparse Hi-C-derived polymer model with the bulk Hi-C-derived one, we computed their genomic overlaps (Section 4). Across all considered downsampling levels, the computed overlaps were consistently higher than a random control built by bootstrapping the model binding site positions (Figure 1H). Statistical significance was assessed using one-sided Mann–Whitney U tests with Benjamini–Hochberg correction for multiple comparisons. Effect sizes, reported as median differences and Cliff’s δ, indicate a consistent and non-negligible separation between real and control distributions across all input levels (Figure 1H, Table S1 and Section 4). These results indicate that the algorithm consistently recovers domain architecture even from sparse input.

Finally, to further evaluate robustness across different input data, we modeled a distinct, 1.5 Mb-wide genomic region (hg19, chr7: 155,400,000–156,800,000) at a ten times higher resolution of 10 kb, using independent bulk Hi-C data from human IMR90 cells [4], generating artificial sparse datasets as above, and obtained similar results (Figure S2). Interestingly, these higher resolution models also enabled the recovery of finer scale structures, such as loop contacts, from sparse data in which these structures were not directly detectable, further supporting the robustness and broader applicability of our method (Figure S2).

Taken together, these results demonstrate that our polymer physics-based approach can extract meaningful structural information even from highly incomplete contact data, supporting its application to single-cell Hi-C data, where sparsity is inherent.

### 2.2. Polymer Modeling from scHi-C Reveals Structural Variability of Domain Organization

Next, we reconstructed 3D chromatin organization in single cells from 100 kb resolution single-cell Hi-C (scHi-C) data in HeLa-S3 cells from [28], focusing on the 15 Mb genomic locus (chr7: 110–125 Mb, hg38) discussed above. To ensure sufficient data coverage within the locus, we selected the top 5% of single-cell matrices with the highest contact counts in the region, resulting in 129 cells for modeling. This selection prioritizes cells with sufficient data coverage while preserving population heterogeneity, as reflected in the convergence of aggregated single-cell data toward bulk features (Figure S3 and Section 4).

These data are characterized by great sparsity, with a percentage of missing contacts in the selected scHi-C contact matrices ranging from 95.77% to 98.49%. Based on our validation on artificially downsampled data discussed above, this level of sparsity falls within the regime where the model can reliably reconstruct structural features, corresponding in the considered dataset to approximately 15–30 contacts per Mb.

**Figure 2:**
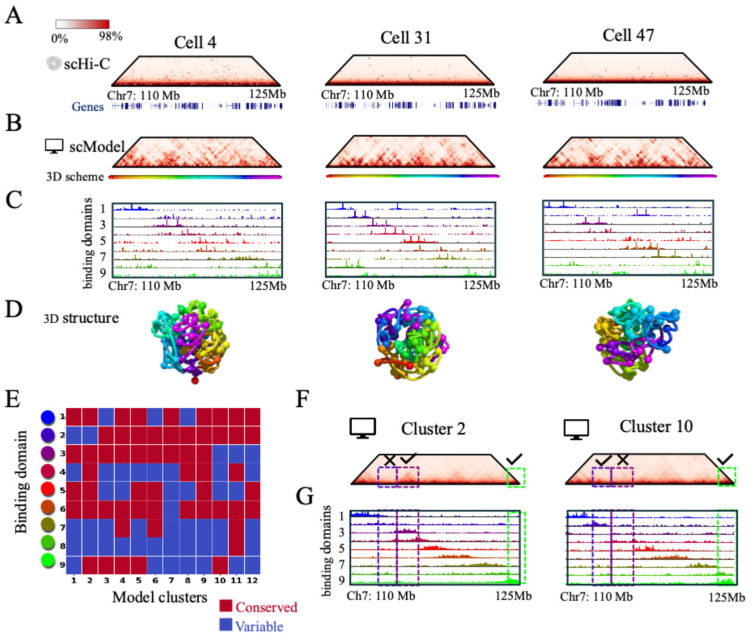
Polymer models from single-cell Hi-C data and their structural variability. (**A**) scHi-C contact matrices of the considered 15 Mb locus (chr7: 110–125 Mb) at 100 kb resolution in HeLa-S3 cell [28] shown for three representative examples of single cells (Cells 4, 31, and 47). The considered cells are indexed based on total contacts count, from highest (Cell 1) to lowest (Cell 129). (**B**) Contact matrices of the single-cell polymer models of the locus inferred from scHi-C data. (**C**) Binding domain profiles of the inferred single-cell polymer models. (**D**) Snapshots of corresponding 3D polymer conformations. The genomic-coordinate-based color bar for the 3D structures is shown below the model matrices in (**B**). (**E**) The inferred single-cell polymer models cluster in 12 different classes based on the overlap between corresponding single-cell (**C**) and bulk (Figure 1F) polymer binding domains (Figure S4 and Section 4). The heatmap summarizes conserved (red) and variable (blue) binding domains (colors) in each of the 12 identified clusters. (**F**) Merged contact matrices (Section 4) from two example clusters, highlighting examples of both conserved (✓) and variable (✗) patterns. (**G**) Merged binding domain profiles (Section 4) from the two example clusters, highlighting conserved (✓) and variable (✗) binding domains that explain the formation of the patterns highlighted in (**F**).

Figure 2A shows three representative examples of experimental single-cell contact maps. Based on such input data, we obtained a different single-cell polymer model for each of the considered cells (Figure 2B–D, Section 4). Despite the sparsity of the input data, the reconstructed model contact matrices are dense and reveal well-defined folding patterns that vary across single cells and partially resemble those seen in bulk data, such as compact, TAD-like domains (Figure 2B). These clear folding features across single cells highlight the model’s ability to reconstruct spatially coherent chromatin folding from highly incomplete experimental input. The inferred binding site profiles (Figure 2C), in turn, partially resemble those derived from bulk HeLa-S3 Hi-C of the same locus (Figure 1F), but also exhibit notable pattern differences between single cells. Example snapshots of the resulting 3D polymer conformations in each single cell are shown in Figure 2D and Figure S4A.

To validate the inferred structures at the level of individual single cells, we quantified the agreement between each scModel-inferred contact matrix and the corresponding experimental scHi-C matrix using the distance-corrected Pearson correlation r′ (Figure S4B). As a control, we generated randomized matrices in which the overall distance-dependent behavior was preserved while the genomic organization of contacts along the locus was disrupted (Section 4). Our models consistently outperformed the controls across all cells, with the per-cell difference Δr′ strictly positive (Wilcoxon signed-rank test, *p* = 6.51 × 10^−23^; median difference = 0.62; Cliff’s δ = 1), indicating that the inferred matrices capture structural features beyond distance-dependent effects.

Next, we systematically investigated the structural variability of single-cell polymer models across the population. To this aim, we first evaluated the similarity of each single-cell polymer model binding domains with those inferred from bulk Hi-C, by computing their genomic overlaps.

Specifically, for each of the nine identified binding domains (colors), we considered the distribution of overlaps between the bulk domain and the corresponding domain in every single-cell polymer (Section 4). These overlap distributions (Figure S4C) were compared to random control distributions generated by bootstrapping the single-cell model binding sites. Statistical comparison revealed that all domains significantly deviate from random expectation after Benjamini–Hochberg correction, with heterogeneous effect sizes across domains (Table S2, Section 4).

So, for each single-cell binding domain, we defined it as conserved, i.e., significantly similar to the corresponding bulk domain, if its specific overlap with bulk is significantly higher than random (exceeds the 90th percentile of the corresponding random distribution), and as variable otherwise (Figure S4D). This analysis revealed non-random combinations of binding domain conservation across the population of single-cell models. Some domains are conserved in most single cells, e.g., domain 6 (brown) is conserved in ~65% of the considered 129 cells, appearing structurally stable across the population. Other domains are much more variable, e.g., domain 8 (green) is conserved in only ~21% of cells.

Next, to identify classes of single-cell polymer models with similar binding domain patterns, we performed a hierarchical clustering of their overlap profiles (Figure S4D). By the Akaike Information Criterion (AIC [45], Figure S4E), we identified 12 distinct clusters of single-cell polymers (Figure S4D), each defined by a characteristic pattern of domain conservation. The centroids for each cluster, i.e., the average overlap of the domains belonging to the given cluster, are shown in Figure 2E, where conserved domains, i.e., those having a significant average overlap (exceeding the 90th percentile of the random control), are represented in red, while variable domains, having non-significant average overlap, are represented in blue. The resulting matrix (Figure 2E) illustrates how different clusters selectively preserve different subsets of bulk-like domains. For example, both cluster 2 and 10 preserve domain 9 (light green); however, cluster 2 also preserves domain 3 (violet) but not domain 2 (dark blue), whereas the opposite is true for cluster 10 (Figure 2E–G). This difference is reflected in the emergence of different contact domains visible in the merged contact matrices of the models in the different clusters (Figure 2F), whose formation is driven by the different arrangement of binding domains in the corresponding polymer models, as highlighted by the representation of the merged binding domains profiles in each cluster (Figure 2G).

In summary, the reconstructed single-cell polymer models have substantial variability in their domain architecture, falling into a small number of different, structurally defined classes. This supports the view that bulk chromatin architecture arises from averaging over a heterogeneous ensemble, reflecting a limited set of distinct domain architectures, rather than from either identical architecture in every cell or entirely unique pattern in each cell.

### 2.3. Validation of Inferred Single-Cell Structures

To validate the inferred single-cell polymer models, we first compared their predictions at the population level against independent bulk Hi-C data. Indeed, bulk Hi-C reflects an ensemble average of single-cell chromatin conformations, providing a natural reference to evaluate whether aggregated single-cell models recapitulate the key structural features observed at the population level.

**Figure 3:**
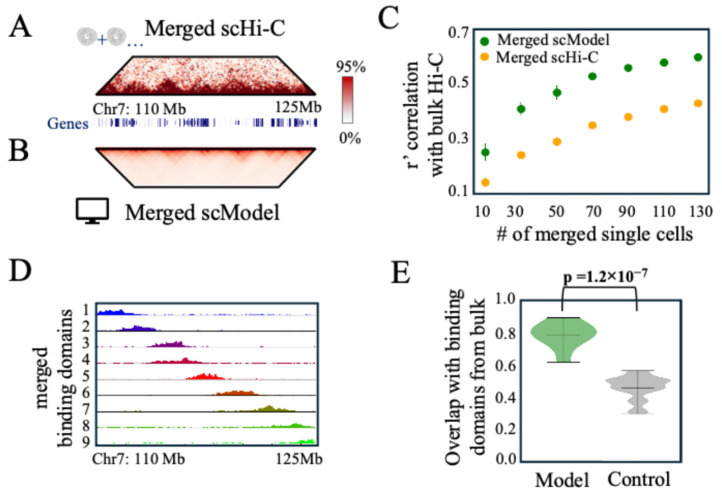
Polymer physics improves sparse scHi-C contact data as validated against bulk Hi-C. (**A**) Contact matrix from merged scHi-C data in HeLa-S3 cells at 100 kb resolution [28]. Its distance corrected correlation with independent bulk Hi-C [44] of the locus is r′ = 0.43. (**B**) Contact matrix from merged single-cell inferred polymer models. Its distance corrected correlation with independent bulk Hi-C of the locus is r′ = 0.60. (**C**) Distance corrected correlation with bulk Hi-C of merged scHi-C contact matrix (orange) and merged model contact matrix (green) as a function of the number of merged single cells. For each number of merged single cell, the plot shows the average correlation obtained from 10 random samples from the full considered set of 129 cells. Error bars indicate standard deviation. (**D**) Binding domains profiles of the merged single-cell polymer. (**E**) Overlap distribution between merged single-cell and bulk derived polymers (green). The distribution of overlaps is significantly higher compared to a random control distribution (gray) obtained by computing overlaps between bulk derived polymer and merged single-cell polymer with bootstrapped binding sites. Statistical significance was assessed using a one-sided Mann–Whitney U test (*p* = 1.19 × 10^−7^). The effect size, quantified by the median difference (0.32) and Cliff’s δ (1), indicates a strong separation between real and control distributions.

By simply aggregating the considered scHi-C data [28], we obtain an experimental merged contact map (Figure 3A) that approximates the population-average contact pattern. This map has a distance corrected correlation of r′ = 0.43 with independent bulk Hi-C [44] of the locus in the same cell line (Figure 1A). However, the merged scHi-C matrix is much noisier than Hi-C, especially at long-range distances. To check if the model can improve the similarity with Hi-C, and consequently predictions of bulk features, we built the predicted contact matrix from the population-averaged polymer model (Figure 3B), obtained by merging the domain profiles of all inferred single-cell polymers (Figure 3D, Section 4). The obtained merged model contact matrix (Figure 3B) exhibits clear domain structures that better resemble the bulk Hi-C map (Figure 1A) compared to the raw merged scHi-C map (Figure 3A), as quantified by the higher distance-corrected correlation (r′ = 0.60 vs. r′ = 0.43).

Next, we checked the performance of the model by varying the number of total merged single cells. To this aim, we randomly selected subsets of single cells of different sizes (from 10 to 129 cells) from the considered total set of 129 cells, and calculated the distance-corrected correlation between the bulk Hi-C matrix and either the obtained merged model or merged raw scHi-C matrix as a function of the number of merged single cells (Figure 3C, Section 4). Interestingly, across all considered subsample levels, the model consistently outperformed the sum of raw sHi-C, and correlations with Hi-C stayed relatively high even when considering very small numbers of single cells. These results highlight the robustness of the model, which can be reliably applied even when only a limited number of single cells are available.

Next, to further validate the inferred single-cell models, we compared their predicted binding domains against an independent polymer model based on bulk Hi-C. Specifically, we compared the binding domains profiles of the merged single-cell polymer model (Figure 3D), with those derived from bulk Hi-C (Figure 1F), by computing their genomic overlaps. We found a remarkable agreement with the bulk polymer, with statistically significant overlaps between corresponding types of binding domains (colors). To assess significance, we compared the model overlaps to a control case in which single-cell binding sites were randomly bootstrapped (Section 4). As shown in Figure 3E, model overlaps, ranging from 0.61 to 0.89, were significantly higher than those found for the random control (one-sided Mann–Whitney U test, *p* = 1.19 × 10^−7^), confirming that the merged single-cell polymer model predicts meaningful domain organization. The effect size was large, as indicated by the median difference (0.32) and Cliff’s δ = 1, reflecting a complete separation between real and control distributions. Finally, as an even more stringent test, we found that the model overlaps were significantly higher than those obtained using a block copolymer model specifically designed to reproduce the main features of the bulk Hi-C matrix (one-sided Mann–Whitney U test, *p* = 0.04, Section 4). Overall, these results show that the polymer models obtained from sparse single-cell data can reconstruct features of chromatin architecture. By inputting missing information and reconstructing structural patterns across single cells, the approach enables us to accurately predict bulk-like genome folding from sparse single-cell data.

Summarizing, our inferred single-cell polymer models have been validated by their accurate prediction of population-level features of chromatin architecture, supporting the view that they can effectively impute missing information and reconstruct structural patterns from sparse single-cell data.

### 2.4. Identification of TAD Boundaries in Single Cells

Building on the observation that polymer modeling can reconstruct global contact patterns from sparse data, we next evaluated whether it can also capture specific, biologically relevant chromatin features, such as TADs in single cells. We compared TAD boundaries predicted directly from raw scHi-C matrices with those obtained from model-derived matrices, and next analyzed their relationship with those identified in bulk Hi-C.

**Figure 4:**
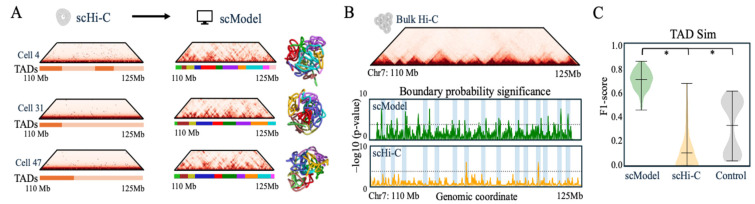
TAD boundary probability across single cells. (**A**) TADs identified from raw scHi-C matrices (left) and from corresponding inferred scModel matrices (right), for three representative single cells (Cells 4, 31, and 47, as in Figure 2). Raw scHi-C matrices are too sparse to reliably identify TADs, whereas polymer modeling enhances structural patterns, making single-cell matrices suitable for TAD calling. Snapshots of 3D structures, with different segments colored according to TADs disposition, are shown next to the scModel matrices (color codes under the corresponding matrices). (**B**) Statistical significance (−log_10_ *p*-value, binomial test with Bonferroni correction) of boundary enrichment across model (green) and experimental (orange) single -cell matrices, calculated per genomic bin. Shaded blue vertical bars indicate 0.3 Mb-wide regions around TAD boundaries called from bulk Hi-C. Bulk Hi-C contact matrix is also shown to help visualization. Dashed horizontal lines represent the significance threshold (*p* < 0.05). Single-cell model boundaries are significantly enriched in correspondence of bulk TADs, whereas experimental single-cell boundaries show limited agreement. (**C**) Distribution of F_1_ scores (TAD similarity metric from ref. [46] comparing single-cell and bulk TAD boundaries. Model single-cell matrices (green) significantly outperform experimental matrices (orange), as well as random control models (gray) built by shuffling single-cell boundaries. Statistical significance was assessed using one-sided Mann–Whitney U tests (ScModel vs. Control: *p* = 9.83 × 10^−55^; ScModel vs. ScHi-C: *p* = 4.94 × 10^−44^). Effect sizes, quantified by the median difference (0.26 and 0.71, respectively) and Cliff’s δ (0.97 and 0.99), indicate a strong separation between distributions. * *p* < 0.05.

As expected, scHi-C data are too sparse and noisy to reliably identify TADs (Figure 4A, left panel, Section 4). Visual inspection of the contact matrices reveals that domain-like patterns are largely absent, and the called TADs appear often randomly positioned rather than aligned with discernible structures. In contrast, the model-reconstructed contact matrices display clear domain structures, enabling precise boundary identification (Figure 4A, right panel). The resulting model 3D conformations illustrate how the different TADs fold into distinct globules. Interestingly, we found a pronounced variability in the TAD boundary number and position from cell to cell, as highlighted by the three representative model single cells shown in Figure 4A, right panel. Quantification across all modeled single cells revealed a broad distribution of boundary counts (Figure S5A). While the average number of boundaries per cell is comparable to that observed in bulk Hi-C data (17.5 ± 1.8 vs. 15), we observed substantial heterogeneity across single cells, with ~5% of cells exhibiting more than 20 boundaries and ~5% fewer than 15 boundaries.

Next, we examined whether TAD boundaries positions in single cells, even though highly variable, tend to occur preferentially at bulk-defined positions as expected [33,47]. To this aim, for each genomic bin, we computed the fraction of single cells in which that bin was called as a boundary. We identified significant boundaries at genomic bins where the frequency of boundary calls across cells was significantly higher than expected under a binomial null model with uniform probability (Bonferroni-adjusted *p* < 0.05; Section 4). Figure 4B shows that modeled single-cell boundaries accumulate at bulk positions, producing statistically significant peaks above the expected baseline, while raw single-cell boundaries exhibit weaker and more diffuse enrichment.

Finally, we quantified the overlap between single-cell and bulk TADs using the TAD Sim F_1_ score [46], which measures the similarity between predicted boundaries in single cells and bulk boundaries by combining precision (the fraction of predicted boundaries matching a bulk one) and recall (the fraction of bulk boundaries recovered among the predictions) into a single harmonic mean. As shown in Figure 4C, F_1_ scores are significantly higher for TADs called from model matrices than for experimental data, and the model consistently outperforms a random control built by randomly shuffling boundaries (one-sided Mann–Whitney U tests, ScModel vs. Control: *p* = 9.83 × 10^−55^; ScModel vs. ScHi-C: *p* = 4.94 × 10^−44^). Effect sizes were large in both comparisons, with median differences of 0.26 and 0.71 and corresponding Cliff’s δ values of 0.97 and 0.99, indicating a strong separation between distributions.

Overall, these results demonstrate that our polymer physics models not only generally improve sparse and noisy scHi-C contact matrices, but also enhance the identification of specific, biologically meaningful features such as TAD boundaries in single cells, supporting the use of polymer physics to bridge the gap between sparse single-cell data and population-level chromatin organization.

### 2.5. Single-Cell Binding Domains Fall into Epigenetic Classes with Distinct Cell-to-Cell Variability

**Figure 5:**
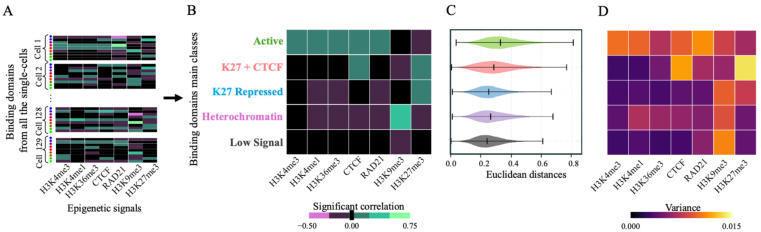
Epigenetic characterization of single cells binding domains reveal heterogeneous classes with distinct variability. (**A**) Scheme illustrating the derivation of epigenetic classes of binding domains. Each binding domain across all single cells (rows) is associated with an epigenetic signature by computing its correlation with key bulk epigenetic factors [48] (columns). The domains are then clustered based on their epigenetic signature in the main epigenetic classes shown in (**B**). (**B**) Heatmap showing the epigenetic signatures of the centroids of the five epigenetic classes identified by hierarchical clustering (Section 4). (**C**) Distribution of pairwise Euclidean distances among single-cell epigenetic signatures within each class, representing intra-class variability. Classes labeled as Active and K27 + CTCF exhibit significantly higher dispersion compared to Heterochromatin, K27 Repressed, and Low Signal classes, reflecting greater cell-to-cell epigenetic heterogeneity (mean *p*-value = 4.74 × 10^−25^, one-sided Mann–Whitney U test; Cliff’s δ = 0.32). (**D**) For each class, variance of correlations of the single-cell binding domains in the class with each epigenetic factor. Active classes display higher variances, particularly across activating histone marks (e.g., H3K4me3).

To investigate the biological nature of the model-inferred single-cell binding domains, we characterized their association with available epigenetic marks at the analyzed locus. Specifically, following our previous approach [13], we computed for each binding domain a vector of Pearson correlations with key bulk epigenetic mark tracks available from the ENCODE database in the HeLa-S3 cell line [48] (Figure S6A). To ensure biological relevance, only correlations significantly higher than those from a random control with bootstrapped binding site positions were retained (Section 4). These correlations define an epigenetic signature for every binding domain across all single-cell polymer models (Figure 5A, Section 4). As shown in Figure S6B, many domains exhibited complex combinations of epigenetic signals, rather than simple associations with individual marks.

Next, we performed hierarchical clustering of these epigenetic signatures (Figure S6B) and determined the optimal number of clusters to be five, based on the Akaike Information Criterion (AIC [45], Figure S6C). Each of the identified statistically distinct epigenetic classes can be characterized by its centroid, defined as the average epigenetic signature among all domains in the class, representing its characteristic pattern of correlation with the considered epigenetic signals (Figure 5B).

The first class, Active, is characterized by positive correlations with transcription-associated histone marks H3K4me3, H3K4me1 and H3K36me3, as well as the architectural proteins CTCF and RAD21, typical of active regulatory regions and expressed gene bodies [49,50,51,52,53].

The second class, K27 + CTCF, is a hybrid class showing concurrent enrichment for H3K27me3 and CTCF, suggesting it marks domain boundaries or regulatory regions at the interface between repressed and active chromatin. Such co-localizations have been proposed to insulate Polycomb domains or contribute to architectural demarcation within facultative heterochromatin [54,55].

The third class, K27 Repressed, is dominated by high correlations with H3K27me3, a hallmark of Polycomb-mediated repression, also reflecting regions subject to the facultative silencing of developmental genes. It is consistent with the Polycomb-repressed chromatin states observed in mammalian and Drosophila genomes [49,56].

The Heterochromatin class is so called because it is enriched for H3K9me3, a signal associated with constitutive heterochromatin and displays low or negative correlations with transcription-related features. It corresponds to densely packed genomic regions, often lamina-associated, involved in transcriptional silencing and structural integrity [55,57].

The last class, named Low Signal, exhibits low correlations across all epigenetic marks, indicating an absence of active or repressive modifications. It aligns with previously described “quiescent” chromatin states, characterized by gene-poor and transcriptionally inert domains [50,53].

Next, we asked how much variability is present within each identified class. To capture global intra-class heterogeneity of the epigenetic signature, we computed for each class the distribution of Euclidean distances between epigenetic signatures of all single-cell binding domain pairs in the class (Figure 5C). Interestingly, Active and K27 + CTCF classes displayed broader, high-values-shifted distributions, indicating higher cell-to-cell variability in their epigenetic profiles compared to K27 Repressed, Heterochromatin and Low Signal classes. To account for differences in sample size across classes, statistical significance was assessed by randomly selecting an equal number of pairwise distances (1000 per group) from the compared classes and then performing one-sided Mann–Whitney U tests. The mean *p*-value across 1000 iterations was 4.74 × 10^−25^. The effect size, quantified by Cliff’s δ (0.32), indicates a moderate but consistent increase in variability in active classes (Section 4).

Analysis of the variance of correlations, separately for each epigenetic factor, around their centroids further confirmed this trend, with Active and K27 + CTCF classes showing consistently higher variance, particularly across activating histone marks (e.g., H3K4me3), compared to K27 Repressed, Heterochromatin, or Low Signal classes (Figure 5D).

These findings support studies revealing that epigenetically active chromatin domains are structurally and functionally more heterogeneous, while repressed or silent domains exhibit greater epigenetic stability across cells [54,55]. Consistently, we also found that binding domains characterized by higher cell-to-cell structural variability (labeled as variable domains, compared to conserved domains, in Figure 2E) exhibit higher epigenetic variability (Figure S7 and Section 4).

Finally, to investigate the contribution of the different classes of binding domains to the locus architecture, we performed a series of in silico mutation experiments. Specifically, for each class, we erased the domains belonging to that class from all single-cell models, computed the mutated merged model and measured its distance-corrected correlation with the bulk Hi-C matrix (Figure S8). As shown in Figure S8A, this analysis revealed class-dependent differences in structural impact. To test their significance, we compared the observed correlation loss for each class to that expected under a control model where we randomly erase the same number of binding domains independent of their epigenetic identity (Figure S8B,C, Section 4). Heterochromatin emerges as the most structurally impactful class: its removal leads to the strongest correlation drop (Δr′ = 0.40), well above the random control. This suggests that domains marked by H3K9me3 play a central role in shaping the overall architecture of the locus, in agreement with previous findings about the stability of constitutive heterochromatin [55]. The two Polycomb-associated classes (K27 Repressed and K27 + CTCF) exhibit a lower but significant loss, suggesting a more localized contribution to folding. Active and Low Signal domains produce the smallest Δr′ drops, likely reflecting two different underlying reasons. Active domains are highly variable across cells (as shown in Figure 5C,D), and their structural contribution may not consistently colocalize across the population, limiting their cumulative structural impact. Low Signal domains, on the other hand, are defined by the absence of strong epigenetic features and likely represent gene-poor, quiescent regions with more limited involvement in 3D organization.

## 3. Discussion

scHi-C data offer valuable insights into chromatin folding at the single-cell level, but their interpretation remains challenging due to extreme sparsity and limited availability of analysis tools, which currently rely on machine learning [33,37,38,58]. Here, we show that a polymer physics-based approach enables the accurate reconstruction of 3D chromatin structures from sparse scHi-C, overcoming the limitations of sparse input data and recovering structural information inaccessible otherwise.

Using artificial sparse data generated by the downsampling of bulk Hi-C [4,44] in the considered 100 kb resolution HeLa-S3 cell and the 10 kb resolution IMR90 cell loci, we showed that our method faithfully reconstructs contact maps and binding site architectures even at low data coverage. Applied to real scHi-C data [28], model-derived structures of the studied HeLa-S3 cell locus showed stronger agreement with independent bulk Hi-C than aggregated raw single-cell contacts at the population level, indicating that modeling effectively imputes missing contacts and restores structural coherence. At the level of domain organization, binding site arrangements inferred from scHi-C were in significant agreement with those obtained from independent, bulk-derived models, further validating the reconstructed structures.

The reconstructed models reveal high cell-to-cell variability in genome architecture. Importantly, our approach identified a dozen distinct clusters of domain architectures, supporting the view that bulk structures emerge from averaging a heterogeneous yet structured ensemble of single-cell conformations [29,31]. Notably, polymer-based imputation enabled the identification of TAD boundaries in single cells, which were found to fluctuate among cells yet converge around bulk positions, consistent with previous reports [33,47].

Another key finding of this work is that single-cell binding domains display epigenetic signatures that fall into distinct classes, consistent with independent chromatin segmentations, showing diverse cell-to-cell heterogeneity. Active and K27 + CTCF classes were associated with higher structural and epigenetic variability across cells, whereas repressed or heterochromatic domains showed greater stability [54,59]. Perturbation analyses further highlighted the prominent role of Heterochromatin in maintaining locus architecture, in line with its established structural role [55,57]. These results provide a mechanistic link between structural variability and epigenetic identity, suggesting that epigenetically active domains contribute to more dynamic aspects of folding, while repressive domains act as stabilizing scaffolds.

**Figure 6:**
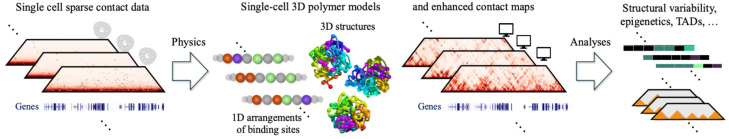
Polymer modeling enables 3D genome reconstruction and data enhancement from sparse single-cell Hi-C. Single-cell Hi-C contact matrices are sparse and noisy, limiting direct inference of chromatin architecture and bioinformatic analyses (**left**). Our approach infers binding sites profiles that drive polymer physics-based reconstruction of 3D genome conformations consistent with scHi-C data (**middle left**), enabling analyses such as single-cell structural variability and epigenetic characterization (**right**). The reconstructed 3D models also yield enhanced contact matrices (**middle right**) improving interpretability and enabling downstream applications such as identification of TADs at the single-cell level (**right**).

In summary, by grounding reconstructions in a physical model of chromatin folding, our approach provides biologically interpretable 3D structures at single-cell resolution, allowing the effective imputation of missing contacts and supporting robust downstream analyses, including the identification of key folding features such as TADs (Figure 6). While this study serves as a proof-of-principle, demonstrating the feasibility and interpretability of polymer physics–based modeling on highly sparse single-cell Hi-C data, the underlying framework is expected to be broadly transferable across genomic scales and experimental conditions as it relies on established physical principles of chromatin folding.

Nevertheless, several limitations remain. Extending from multi-megabase loci to whole-genome reconstructions and at higher resolution will require improved computational efficiency. Integration with machine learning approaches could enhance scalability and accuracy by combining complementary strengths in physics-based modeling and data-driven inference [33,34,35,37]. Moreover, while we validated our models against independent bulk and epigenetic data, experimental orthogonal validation at the single-cell level, for example via imaging [49], would provide further support.

Despite these challenges, our results highlight the power of physics-based modeling to extract meaningful information from sparse single-cell contact maps. Future extensions to whole-genome reconstructions, integration with machine learning, and adaptation to other sparse datasets such as promoter capture Hi-C [53] and single-cell multi-omics [31,33] will further expand its scope. These developments will enhance the applicability of the method and deepen our understanding of how genome folding underpins dynamic and heterogeneous cellular states, thereby linking 3D genome architecture with epigenetic regulation, gene expression, and cell function.

## 4. Methods

### 4.1. Loci and Datasets

The analyzed 15 Mb genomic locus on chromosome 7 in the human HeLa-S3 cell line has coordinates chr7: 110,000,000–125,000,000 (hg38). Bulk Hi-C data of the locus were obtained from [44]. In particular, we downloaded Hi-C maps in *.mcool* format and extracted the contact matrix for the considered locus at 100 kb resolution. To correct for experimental biases, we next applied Knight&Ruiz (KR) normalization [60] using the Python package *cooler* [61] (Python version 3.11.14, cooler version 0.10.4).

The coordinated of the analyzed 1.5 Mb-wide genomic region on chromosome 7 in IMR90 cells are chr7: 155,400,000–156,800,000 (hg19). Bulk, in-situ Hi-C data at 10 kb resolution of the locus in IMR90 cells with KR normalization were taken from [4].

Single-cell Hi-C data, including raw contact pairs data and processed cellular indices, were retrieved from Ramani et al. [28]. In this dataset, single cells had already undergone barcode demultiplexing and quality-control filtering, including removal of low-coverage cellular indices, low cis/trans ratio cells, and ambiguous species assignments in the species-mixing experiments. Only cellular indices confidently assigned to HeLa-S3 cells were retained for the present analysis. Contacts within the chosen locus were then binned at 100 kb resolution to generate single-cell contact matrices.

ChIP-seq data were obtained from the *ENCODE Consortium* (https://www.encodeproject.org/, accessed on 13 May 2026, [48]) for seven key chromatin-associated factors in HeLa-S3 cells, comprising active marks (H3K4me3: accession code ENCFF432PYK; H3K4me1: ENCFF860GGZ; H3K36me3: ENCFF157RUQ), repressive marks (H3K9me3: ENCFF658AOG; H3K27me3: ENCFF186NYC), and architectural proteins (CTCF: ENCFF179RSE; RAD21: ENCFF357XMG). Signal tracks were binned at 100 kb resolution by using the *map* command from the *bedtools* suite (v2.31.1) [62].

### 4.2. SBS Polymer Model and PRISMR Inference

To model the 3D organization of the considered genomic loci, we employed the Strings & Binders Switch (SBS) polymer model of chromatin [6,7,11]. In this model, a chromatin region is represented as a self-avoiding walk (SAW) polymer chain of beads. Some beads act as binding sites and interact only with specific diffusing molecular binders, while others are inert, non-interacting beads. Binding sites are characterized by their specific binding affinities. The system is governed by the binder concentration and binding energy, and their values are chosen to produce compact globule conformations. Specific interactions between distinct types of binding sites (referred to as colors) and their cognate binders drive the organization of chromatin into complex 3D structures through microphase separation.

To infer the SBS model of a specific locus, i.e., the optimal binding site arrangement along the SBS polymer that best reproduces the folding of the considered loci, we applied the PRISMR algorithm, which is fully described in ref. [40], and we briefly summarize here. PRISMR takes as input a contact probability matrix, e.g., from experimental Hi-C data, and searches for the minimal number of colors, n, and their arrangement along the polymer that best recapitulates the input matrix. To take into account that more binding sites, interacting with one or more different factors, can locate in the same genomic window at the given Hi-C resolution, the model allows to locate up to r beads in each window. The polymer model optimization is made via an iterative Simulated Annealing (SA) Monte Carlo procedure, which minimizes a cost function *H* made of two terms. The first term, *H*_0_ is a standard mean squared error between the experimental and model derived contact map, which quantifies the discrepancy between experimental and model-predicted contact probabilities. The second is a Bayesian term, H_λ_, that penalizes the addition of binding sites in the polymer, in order to reduce overfitting. H_λ_ is weighted by a regularization parameter λ ≥ 0 that represents the cost of adding a single binding site along the polymer. In this way, we account for both the necessity of fitting the input data well and to avoiding overfitting. To start the PRISMR procedure, we first initialize the SBS polymer in a random configuration, obtained by randomly assigning a color to each polymer bead. Next, we optimize the model via a standard iterative SA procedure [63,64]. In each SA move, a polymer bead is randomly selected, its color randomly modified, the resulting polymer’s average contact map recalculated, the corresponding cost function evaluated, and this then compared with that from the previous step to determine whether the change is accepted or rejected. The process is repeated iteratively until convergence of the estimated optimal value of the cost function, i.e., until its changes are within 0.1%. To identify optimal values of the model parameters n, *λ*, r, the entire procedure is performed multiple times using different initial polymer configurations and parameter settings as described below.

### 4.3. Modeling from Bulk Hi-C

To estimate the optimal parameters, n, *λ*, r, we followed the machine learning approach developed in [12]. Specifically, for the bulk Hi-C data of the two considered loci, the contact matrix was randomly split into a training set made of 70% of matrix elements and a complementary 30% test set. PRISMR was run on the training set and model performance was evaluated on both subsets using the score function *H*_0_. As done in [12], to take into account genomic distance effects characterizing bulk Hi-C data, we used a scaled cost function where we divided each term of *H*_0_ by the mean Hi-C contact value at the corresponding genomic distance during the SA procedure. The optimal number of colors, *n*, was selected as the value minimizing *H*_0_ on the test set. The regularization parameter, *λ*, and the bead-to-bin ratio, *r*, were estimated in a similar way, by finding the values that minimize *H*_0_ while keeping the other parameters fixed. Specifically, we found *n* = 9, *λ* = 1 × 10^−5^, *r* = 20 for the considered 100 kb resolution HeLa-S3 cell locus (Figure 1A,B,F), and *n* = 3, *λ* = 1 × 10^−5^, *r* = 20 for the 10 kb resolution IMR90 cell locus (Figure S2A–C).

Finally, for each locus we ran 20 independent SA simulations from different initial polymer configurations, with these fixed values for the parameters *n*, *r* and *λ*, and selected the one achieving the lowest value of the cost function as the optimal SBS polymer model (Figure 1B,F).

The similarity between experimental and model-derived contact matrices was quantified using the distance-corrected Pearson correlation coefficient *r*′ [40], excluding genomic distances below 30 kb for 10 kb resolution data and below 0.7 Mb for 100 kb resolution data to correct for outliers. *r*′ is the Pearson correlation computed on contact matrices where from each element the mean value of the diagonal to which it belongs is subtracted and has been used to put aside the distance-dependent decay of the contact probability which tends to dominate in the usual Pearson correlation.

### 4.4. Modeling from Artificial Sparse Hi-C

To evaluate the robustness of the PRISMR inference procedure under increasing data sparsity, we applied it to downsampled versions of the bulk Hi-C contact matrix at 100 kb resolution. Downsampling was performed by randomly retaining a specified fraction of matrix entries (corresponding to pairs of genomic loci), leaving their contact values unchanged, while setting all remaining entries to zero. For example, a sparsity level of 80% results in a matrix in which 20% of entries are non-zero values. For each downsampling level, 20 independent replicates were generated using different random seeds. Examples of the generated artificial sparse Hi-C matrices are shown in Figure 1C and Figures S1 and S2D. This procedure results in a uniform sparsification of the Hi-C matrix, which provides a controlled and unbiased strategy to assess the impact of sparsity on model performance without introducing additional assumptions on the structure of missing data.

Each artificial sparse matrix was used as an input to the SA procedure algorithm, using the same scaled cost function and the same parameters *n*, *λ* and *r* inferred from the full bulk Hi-C dataset.

To better deal with high sparsity, for percentage of downsampling less than 10%, we replaced zeroes in the input matrices with the theoretical contact probability between inert sites, as described in Section 4.6 below.

For each downsampling level, a polymer model is inferred by our SA procedure from all 20 different random replicates, and, among these, the optimal polymer model was selected as the one whose predicted contact matrix, computed in a mean-field approximation [40], achieved the highest distance-corrected Pearson correlation r′ with the original full-resolution bulk Hi-C matrix (Figure 1D,G and Figures S1 and S2E,F).

To evaluate the model accuracy across input Hi-C sparsity levels, we computed the mean and standard deviation of r′ values over 20 replicates for each down-sampling level (Figure 1E and Figure S2E). We found that r’ increases with increasing input Hi-C and reaches a plateau, identified as the value where it exceeds 90% of its spanned range, at approximately 50% of input Hi-C. While this is an optimal threshold, we found that r’ is significant across the whole considered range of tested sparsity levels. To assess significance, we performed the same analysis on randomized model contact matrices as a control case, in which we shuffled contact values within each diagonal, thereby preserving the genomic distance-dependent decay of contact probabilities while removing locus-specific structural patterns (Figure 1E).

To further assess the reliability of the polymer models inferred at each level of input Hi-C sparsity, we compared the binding domains from these models with those inferred from the full bulk Hi-C. To this aim, for each pair of binding domains, we computed their genomic overlap [28] and compared it to a null distribution obtained by randomly bootstrapping the binding sites positions 50 times (see Section 4.8 below). Statistical differences were evaluated using one-sided Mann–Whitney U tests. To account for multiple comparisons across downsampling levels, *p*-values were adjusted using the Benjamini–Hochberg procedure. In addition to statistical significance, effect sizes were quantified using the median difference between real and control overlaps and Cliff’s δ (Table S1, Figure 1H).

### 4.5. Processing of scHi-C Data

scHi-C data at 100 kb resolution for the HeLa-S3 cell locus were obtained from [28] as described above and converted from hg19 to hg38 using the UCSC *liftOver* tool (https://genome.ucsc.edu/cgi-bin/hgLiftOver, accessed on 13 May 2026; [65]).

From the full set of 2574 processed cells, we selected the top 5% with the highest number of contacts within the target locus (Figure S3A). This resulted in 129 cells with at least 250 contacts in the locus. This filtering ensured that polymer modeling was performed only on single cells with sufficient coverage in the locus (chr7: 110–125 Mb), independently of genome-wide contact density. The 5% threshold was empirically chosen as a trade-off between maximizing locus-specific coverage and retaining a sufficiently large number of high-quality single-cell matrices for modeling.

We also measured the distance-corrected Pearson correlation r′ between the bulk Hi-C matrix and the merged contact matrix from the top single-cell Hi-C matrices, ordered by contact number in the selected locus (Figure S3B). The correlation increases rapidly with the number of merged cells and reaches ~70% of the maximum achievable correlation with bulk already with the top 5% (129 cells), confirming that the selected single cells were enough to obtain a good description of the locus. Beyond this point, the curve reaches a plateau and eventually starts decreasing. Adding lower-coverage cells progressively reduces the correlation, indicating that these cells contribute predominantly noise to the aggregate signal. A more detailed view of the top 10% of single cells (Figure S3C) further supports this conclusion, showing that including up to the top 10% of cells (254 cells) yields only a modest increase in correlation (r′ ≈ 0.48), while the top 5% already achieves r′ ≈ 0.43 (~90% of the maximum), supporting this threshold as an effective trade-off between data coverage and population size. Contact pairs from the selected cells were finally binned at 100 kb resolution to generate symmetric contact matrices.

### 4.6. Modeling from scHi-C

To model the chromatin structure of individual cells, we applied the PRISMR inference framework to each of the 129 scHi-C contact matrices at 100 kb resolution, selected as described above.

To deal with sparsity in the single-cell matrices, we introduced a weak, distance-dependent baseline contact probability for missing entries based on polymer physics. Specifically, zero entries were filled with the average contact probability between inert polymer beads at the corresponding genomic distance, as expected for a classical self-avoiding walk (SAW) polymer. This baseline probability, $P_{open}\left(s\right)$, was computed via Molecular Dynamics simulations of an SBS homopolymer made of inert (i.e., non-interacting) beads, followed by computation of the average contact probability P(s) between pairs of beads separated by a genomic distance s [11]. At large distances, this probability follows a power-law scaling $P_{open}(s)∼{\left(\frac{1}{s}\right)}^{\alpha }$, with α = 2.1, consistent with the expected behavior of the randomly folded, open SAW polymer model. Importantly, to calibrate this baseline to the range of experimental data, we scaled $P_{open}\left(s\right)$ by a normalizing factor, obtained by comparing $P_{open}\left(s\right)$ with the empirical contact probabilities observed in the scHi-C matrices. In particular, we computed the empirical contact probability observed in single-cell data at short genomic distances (up to 1 Mb), by averaging over all single cells, and found that the empirical values are centered around ~30% of the theoretical curve. Based on this observation, we rescaled $P_{open}\left(s\right)$ by a factor of 0.3 before using it for zero-filling. As a result, the filled values introduce only a smooth distance-dependent decay along each matrix diagonal, without adding any locus-specific structural information. These values are applied only to missing entries and do not modify observed contacts. In the optimization procedure, filled and observed entries are treated identically; however, due to their reduced magnitude, the filled values act as a weak baseline and do not dominate over experimentally observed contacts. This approach provides a minimal physics-based background that stabilizes the inference while preserving the structural signal present in the data.

Next, for each single-cell input matrix, five independent SA simulations were run from different initial polymer configurations, using the bulk Hi-C-derived parameters *n* = 9, *λ* = 1 × 10^−5^ and *r* = 20, and the optimal polymer model was selected as the one achieving the lowest value of the cost function *H*. The obtained bead sequence was then used to perform large-scale molecular dynamics simulations within the SBS framework.

To assess sensitivity to these parameter choices, we repeated the single-cell inference over a range of n and λ values, varying one parameter at a time. For each condition, we evaluated the merged-scModel correlation with bulk Hi-C (as in Figure 3C), the mean overlap between the merged single-cell polymer and the bulk-derived polymer (as in Figure 3E), and the TAD similarity F_1_ score (as in Figure 4C). This analysis, reported in Figure S9, shows that all three metrics are largely stable under variations in λ, while a moderate dependence on n is observed, with a saturation around n = 9, supporting the robustness of the main findings and the choice of bulk-derived parameters.

To evaluate model performance at the single-cell level, we computed the distance-corrected Pearson correlation (r′) between each inferred contact matrix and the corresponding experimental scHi-C matrix. As a control, randomized matrices were generated by shuffling contact values within each diagonal of the inferred matrices, thus preserving genomic distance-dependent decay while removing locus-specific structure. Differences between real and control correlations were assessed using a Wilcoxon signed-rank test (*p* = 6.51 × 10^−23^), and effect sizes were quantified using the median difference (0.62) and Cliff’s δ = 1 (Figure S4B).

### 4.7. Molecular Dynamics Simulations

We implemented the SBS polymer model within the open source, highly parallelized molecular dynamics software LAMMPS, version 30 July2016 [66]. For each-single cell, the system consists of a fully flexible linear polymer chain with N = 3000 beads and 1500 binders, both represented as spherical particles with diameter σ = 1 and mass m = 1 in dimensionless units. The chain connectivity is enforced using the finitely extensible nonlinear elastic (FENE) bond potential between the consecutive beads, with a maximum extension R_0_ = 1.6σ and a spring constant K_FENE_ = 30 K_B_T/σ^2^. The self-avoidance between any pair of beads is modeled using the fully repulsive Weeks–Chandler–Andersen (WCA) potential, which is also applied to binder–binder interactions. The specific and non-specific binding affinities between binding sites and their cognate binders are modeled using a truncated and shifted attractive Lennard–Jones potential with interaction strength ε and cutoff r_c_. For simplicity, we set ε = 3.15 K_B_T and r_c_ = 2.5σ for all specific interactions, and ε = 8 K_B_T and r_c_ = 1.3σ for all non-specific interactions. For these binding affinity values, the binder concentration lies above the critical threshold associated with the coil–globule phase transition. As a result, thermodynamic equilibration favors globular polymer conformations.

Starting from an initial configuration of SAW polymer chain conformation and randomly distributed binders within a cubic simulation box, both the beads and the binders evolve according to the Langevin dynamics. The equations of motion are integrated numerically using the Velocity–Verlet algorithm in LAMMPS within NVE ensemble, with an elementary time step dt = 0.012, a friction coefficient γ = 0.5, and temperature T = 1.0. The system is equilibrated for more than 5 × 10^7^ time steps. Subsequent configurations are stored over 5 × 10^6^ time steps with a sampling frequency of 5 × 10^4^ time steps. For each polymer model, five independent trajectories are simulated, and the full ensemble of stored 3D conformations is used to obtain reliable statistics. Note that thermal fluctuations induce conformational heterogeneity within the ensemble of stored globular conformations.

Using all stored configurations, we computed the average contact matrix. For a given 3D conformation, a pair of beads is considered to be in contact if their Euclidean distance is less than a threshold distance 7.5σ.

Repeating this calculation over all bead pairs yielded the contact matrix for each conformation. Averaging across all such matrices produced the final average contact matrix.

Representative examples of single-cell contact matrices, corresponding polymers and 3D conformations are shown in Figure 2A–D and Figure S4A.

Visualization was performed using the Matplotlib package in Python (version 3.10.15) for contact matrices and polymers, and the POV-Ray software (version 3.7) for rendering 3D conformations.

### 4.8. Characterization of Binding Domains

To quantify the similarity between pairs of binding domains (colors), we measured their genomic overlap [40], defined as the cosine similarity between the vectors containing abundances of binding sites across genomic windows of the two domains.

Specifically, to match the colors of each single-cell model with those of the bulk model, we measured the genomic overlap between all the possible pairs of colors in the considered single-cell and bulk model. We then assigned, in an exclusive way, a given color in the considered single-cell model with the most overlapping one in the bulk. The same procedure was also applied to polymer models obtained from downsampled bulk matrices, ensuring consistent color assignments across all datasets.

To assess the significance of these color-to-color correspondences, we generated a null distribution by randomizing the genomic positions of beads while preserving the total number of beads per color for each polymer. This bootstrapping was repeated 50 times per cell, and for each random polymer the same color assignment was performed against the bulk model. The resulting distributions of random overlap scores were compared to the true overlaps across single cells (Figure S4C). The plot in Figure S4C highlights that true overlaps are generally different from random, showing broader distributions and more often shifted toward higher values. A broader distribution indicates a variability across single cells, with higher overlaps corresponding to similar arrangement between single-cell and bulk binding sites, and lower overlaps indicating a greater rearrangement of binding sites. For each color, the overall deviation of the distribution from the random control was assessed using two-sided Mann–Whitney U tests, with *p*-values adjusted for multiple comparisons using the Benjamini–Hochberg procedure. Effect sizes were quantified using the median difference between real and control overlaps and Cliff’s δ for each domain (Figure S4C and Table S2).

Next, to identify domains consistently maintained across the population of cells, we classified each color in a single-cell polymer as conserved or variable based on its similarity with the bulk. Using the aligned color profiles described above, we compared the overlap of each domain with its bulk counterpart to a null distribution generated by randomizing bead positions while preserving the total number of beads per color. A domain was considered conserved if its similarity to the corresponding bulk color exceeded the 90th percentile of the corresponding null distribution (Figure S4C); otherwise, it was labeled as variable. This yielded a binary conservation matrix (9 domains × 129 cells), used for visualization (Figure S4D).

Finally, to investigate the diversity of binding domain arrangements across single cells, we performed clustering based on the overlap profiles between each single-cell polymer and the bulk model. Each single-cell polymer yields nine overlap values, one per domain, quantifying the similarity with the corresponding bulk domain after alignment described above.

These values were arranged into a 9 × 129 matrix, where each row corresponds to one of the nine structural domains defined in the bulk and each column to a single cell. This matrix was used as the input for hierarchical clustering using Ward linkage and Euclidean distance as the metric, implemented in the *scipy.cluster.hierarchy* Python module (Scipy version 1.17.0). The optimal number of clusters K was selected by minimizing the Akaike Information Criterion (AIC, [45]), which reached its minimum at 12 clusters (Figure S4E). Specifically, the AIC function showed a broad minimum plateau for 10 ≥ K ≥ 14. We therefore selected K = 12, centered within this stable regime. The resulting clusters contain between 8 and 16 cells each (8, 16, 12, 9, 15, 10, 9, 12, 9, 10, 8 and 11 single cells for each cluster from 1 to 12 respectively).

Each resulting cluster represents a subpopulation of single-cell polymers sharing similar overlap profiles. To summarize the obtained structural clusters, we defined a cluster centroid by averaging the nine overlap values across all polymers in the cluster. These centroid vectors were then binarized based on the same null distribution used for individual domain assignments: a domain was marked as conserved in the centroid if its average overlap exceeded the 90th percentile of the corresponding random distribution defined as above, and as variable otherwise.

This clustering approach revealed the presence of reproducible structural subgroups within the population, each characterized by a distinct pattern of conserved and variable domains (Figure S4D and Figure 2E). To summarize the binding domains arrangements and the contact patterns captured by a given cluster of polymers, we also constructed a merged polymer model of each cluster and its associated contact matrix. The binding domains of the merged polymer model of a cluster are obtained by summing the binding sites of each domain from all polymers in the cluster across the locus. Their contact matrices are then computed in the PRISMR mean-field approximation [40] (Figure 2F,G).

### 4.9. Merged Single-Cell Model

To reconstruct population-level chromatin architecture from sparse single-cell Hi-C data, we aggregated the domain profiles of all 129 PRISMR-inferred single-cell polymers. Specifically, for each binding domain type (i.e., each color), we summed in each 100 kb genomic window the binding sites of that type from all single-cell polymers. To consider inert beads, we added a fraction of those in each genomic window in proportion to their total number across single-cell models.

The obtained population-averaged polymer model (merged scModel) is shown in Figure 3D, where the y-axis reporting domain abundance goes from the 20th to the 100th percentile to better visualize domains structures.

To assess how well this merged polymer recapitulates population-level contact patterns, we computed its contact matrix using the mean-field approximation adopted in PRISMR [40] (Figure 3B). For comparison, we also constructed a population-averaged experimental contact matrix (merged scHi-C) by summing the 129 raw single-cell Hi-C contact matrices (Figure 3A). Next, we computed the distance-corrected Pearson correlation r′ of both merged scModel and merged scHi-C matrices with the bulk Hi-C. This analysis was repeated for increasing numbers of randomly selected single cells, from 10 to 129, and r′ values were averaged over 10 random subsets per size (Figure 3C). Error bars represent standard deviations across replicates.

Additionally, we checked that a different strategy to build a merged model contact matrix by directly averaging the model contact maps of the 129 single cells produced similar results, confirming the robustness of the approach.

We then evaluated the genomic overlap between the nine colors of the merged polymer model and those of the bulk-derived polymer model. All overlaps, ranging from 0.61 to 0.89, were significantly above null expectations obtained by bootstrapping the merged polymer 50 times (randomizing bead positions per color; Figure 3E, Mann–Whitney U test, one-sided, *p* = 1.2 × 10^−7^, median difference = 0.32, Cliff’s δ = 1).

As an additional control, we also constructed a block copolymer model of the locus based on the bulk domain structure. This model is built by using the same number of beads and colors as PRISMR-inferred models of the locus, but assigning unique colors in contiguous, non-overlapping blocks to mimic the TAD-like structure of the bulk Hi-C data. Despite being designed to reproduce bulk-level contact features, the block copolymer showed significantly lower overlaps with the bulk Hi-C derived polymer with respect to the merged polymer.

### 4.10. Identification of TADs

To identify TAD boundaries in both bulk and single-cell contact matrices, we implemented an insulation score-based strategy. Given a contact matrix, the insulation score at each bin was computed as the average contact frequency within a diamond-shaped window with an apex on that bin [67,68]. Local minima of this insulation score trace were then defined as candidate TAD boundaries, using the Python *scipy.signal.argrelextrema* function (Scipy version 1.17.0) with a tunable order controlling smoothness. This parameter defines the number of neighboring bins that must have higher insulation scores for a point to be considered a local minimum. For instance, an order of 5 requires that a minimum be lower than at least 5 bins (500 kb) on each side.

We explored a range of parameters spanning window sizes from 0.3 Mb to 1.5 Mb in 0.1 Mb increments and order values of 3, 5, 7, and 9. For each 100 kb genomic bin, we measured the number of times it was identified as a local minimum in each cell across these parameters. We then selected as final boundaries the bins for which this number exceeded the global 90th percentile of the distribution across all bins and cells. To further remove spurious boundaries in adjacent bins, we applied a filtering step whereby boundaries within a distance of 0.3 Mb from each other were considered as a single boundary with their average position. As a consequence of this merging procedure, the resulting boundary coordinates are no longer constrained to the original 100 kb bins and to better represent these merged positions, boundary coordinates were mapped onto a finer 50 kb grid for the following boundary enrichment analysis.

This strategy was applied consistently across the bulk Hi-C, single-cell model, and raw scHi-C contact maps. Representative examples of TAD calling from raw and modeled single-cell matrices are shown in Figure 4A. To assess variability in domain segmentation, we also computed the number of boundaries identified in each individual cell. The resulting distribution of the number of boundaries identified per cell is shown in Figure S5A. The modeled cells show a consistent average of 17.5 ± 1.8 boundaries, close to the bulk value of 15, while experimental cells show low and highly variable counts (2.2 ± 2.8).

### 4.11. Boundary Enrichment

To quantify whether specific genomic positions are recurrently identified as boundaries across the single-cell population, we performed a bin-wise enrichment analysis on both model and experimental single-cell matrices. To this aim, the HeLa-S3 cell locus was divided into 300 bins of 50 kb (from 110 to 125 Mb), and for each single cell, a binary vector was created indicating the presence (1) or absence (0) of a boundary at each bin.

For each bin, we then assessed whether the observed number of boundaries exceeded the frequency expected by chance under a binomial null model. Specifically, we used the *scipy.stats.binom.sf* function in Python to compute the survival function, i.e., the probability *p* of observing *k* or more boundaries out of *N* cells, given the average boundary frequency across all bins in the population. Under the null hypothesis, the total number of boundary calls observed across all cells is fixed, and each boundary is assumed to occur independently with equal probability at any genomic position. To correct for multiple testing, we applied a Bonferroni correction by dividing the significance *p*-value threshold (0.05) by the total number of bins (300).

The resulting −log_10_(*p*) values are shown in Figure 4B, where statistical enrichment is plotted for each bin along the genomic locus, for both model and experimental matrices. The positions of TAD boundaries identified in bulk Hi-C are overlaid as shaded blue regions, each spanning a ±0.15 Mb interval (i.e., 0.3 Mb total) around the boundary center.

### 4.12. TAD Similarity Evaluation

To quantify the overall agreement between single-cell and bulk TAD structures, we computed the F_1_-score between the boundary set predicted in each single cell and that derived from the bulk Hi-C matrix, following the approach from ref. [46]. Specifically, a predicted single-cell boundary was counted as a true positive if located within a matching tolerance threshold of 0.3 Mb from a bulk boundary, with each bulk boundary matched at most once for the considered cell. The resulting precision and recall were then used to compute the F_1_-score (Figure 4C).

The matching tolerance threshold of 0.3 Mb was chosen based on an empirical evaluation of how the F_1_-score varies with increasing tolerance between 0 and 1 Mb. Specifically, we observed that the population-averaged F_1_-scores for both modeled and experimental data exhibited an inflection point (“elbow”) around 0.3 Mb, indicating a balance between sensitivity and specificity (Figure S5B). Nearby values (e.g., 0.4 Mb) yield very similar results, demonstrating that the conclusions are robust with respect to the exact choice of the tolerance parameter.

To assess the statistical significance of these similarity values, we compared the distribution of single cell’s F_1_-scores to two control models. The first control model was generated by sampling 10 random boundary vectors of the same length as the observed one in each single cell, uniformly across the locus (Figure 4C). The second was constructed by randomly generating 10 vectors of the same length as the bulk boundaries and comparing them to the cell’s boundary vector.

Statistical significance was assessed against the two different control models using one-sided Mann–Whitney U tests, which yielded highly significant *p*-values (*p* = 9.83 × 10^−55^ and *p* = 4.94 × 10^−44^ respectively). Effect sizes were additionally quantified using the median difference (0.26 and 0.71, respectively) and Cliff’s δ (0.97 and 0.99, respectively).

### 4.13. Epigenetic Characterization of Single Cell Binding Domains

To characterize the epigenetic landscape associated with the binding domains of the inferred single-cell models, we computed an epigenetic signature for each domain by correlating its spatial profile with seven bulk epigenetic signals from the *ENCODE* database [48]. These signals included active marks (H3K4me3, H3K4me1, H3K36me3), architectural proteins (CTCF, RAD21), and repressive marks (H3K9me3, H3K27me3), all binned at 100 kb resolution across the HeLa-S3 cell locus (Figure S6A). For each binding domain across the 129 single-cell polymer models, we computed the Pearson correlation of the domain abundance across 100 kb bins in the locus with each of the seven epigenetic tracks, after z-score normalization of the latter, obtaining one epigenetic signature per domain.

To ensure the significance of those correlations, we generated 100 randomized control polymers per single cell by bootstrapping the binding sites of each domain across the genomic bins while preserving their total number. For each random domain, correlations with the epigenetic signals were computed, generating a null distribution per signal. Correlation values falling above the 80th percentile or below the 20th percentile of the corresponding null distribution were then retained as significant.

Using these thresholds, 4440 domain-signal entries out of 8127 (~54% of the total) were retained as significant. More stringent thresholds of 90th/10th and 95th/5th percentile reduced the number of retained entries to 2934 (~36%) and 1976 (~24%), respectively. While the overall large-scale epigenetic organization remained qualitatively consistent, stricter thresholds progressively suppressed biologically meaningful combinatorial signatures, particularly those involving CTCF. The adopted 80th/20th percentile thresholds were therefore selected as a trade-off between robustness and biological interpretability.

The resulting (129 × 9) × 7 matrix of statistically significant correlations is displayed in Figure S6B.

Next, to identify recurrent patterns within the domain-level epigenetic signatures, we performed hierarchical clustering on this correlation matrix. Pairwise distances between domain signatures were computed using the Euclidean metric, and clusters were defined using Ward linkage. The optimal number of clusters K was determined by minimizing the Akaike Information Criterion (AIC, [45]) across increasing values of the number of clusters, resulting in five epigenetic classes (Figure S6C). The AIC function showed a well-defined minimum at K = 5. The resulting epigenetic classes contain 174 (Active), 202 (K27 + CTCF), 179 (K27 repressed), 175 (Heterochromatin), and 431 (Low Signal) single-cell binding domains, respectively.

The centroid profile of each class was then computed by averaging the epigenetic signatures of its assigned domains. To assess the significance of these centroid values, we compared each centroid entry to the distribution of the corresponding centroid values obtained by repeating the entire clustering procedure on 100 randomized single-cell polymers generated by bootstrapping as described above. Values falling above the 80th percentile or below the 20th percentile of the control distribution were retained as significant and shown in Figure 5B.

The five resulting epigenetic classes (Figure 5B) were named based on their characteristic correlation profiles with the epigenetic signals: Active, K27 + CTCF, K27 Repressed, Heterochromatin, and Low Signal.

### 4.14. Quantification of Epigenetic Classes Variability

To assess the degree of epigenetic heterogeneity within each of the five identified classes, we first computed pairwise Euclidean distances between correlation profiles of the domains within each class. The distributions of these distances were visualized using violin plots (Figure 5C), providing a global measure of intra-class variability in epigenetic signatures.

To test for statistically significant differences in variability, we compared the pairwise distance distributions of epigenetically active classes (Active and K27 + CTCF) versus the remaining classes (K27 Repressed, Heterochromatin, and Low Signal). To account for unequal sample sizes across epigenetic classes, we implemented a subsampling strategy. Specifically, we randomly sampled an equal number of pairwise distances (1000 per group) from the compared classes and repeated this procedure over 1000 iterations. For each iteration, statistical significance was assessed using a one-sided Mann–Whitney U test. The mean *p*-value across iterations is 4.74 × 10^−25^. Effect size was quantified using Cliff’s δ (0.32).

These results indicate that domains assigned to active classes are markedly more variable in their epigenetic profiles than those in repressed or quiescent classes.

To complement this analysis with a signal-specific measure of heterogeneity, we computed the variance of correlation values across all domains in each class, separately for the seven epigenetic factors. This yielded one variance value per factor per class (Figure 5D), reflecting how consistently each individual signal characterizes domains within the same cluster.

### 4.15. Epigenetic Characterization of Structural Clusters

To investigate the relationship between the structural and epigenetic similarity of binding domains, we analyzed the epigenetic signature of the structural clusters of single-cell polymer models (Figure 2F,G) discussed in the Section 4.8.

As a reference, we also computed the epigenetic signatures of the nine binding domains identified in the bulk polymer (Figure S7A).

For each structural cluster, we computed the epigenetic signature of its binding domains, i.e., correlations of each domain with the seven considered epigenetic tracks and calculated the intra-cluster variance of these correlations across the single-cell polymers belonging to the cluster (Figure S7B). These per-domain variance values quantify the heterogeneity of the epigenetic signal within each structural cluster. In this representation, domains were labeled as conserved or variable depending on their genomic overlap with the bulk polymer, as defined in Figure 2E.

To test if conserved domains across cells also have more stable epigenetic signatures compared to variable domains, we pooled intra-cluster variances from all clusters and grouped them by their conserved/variable label. Statistical comparison between the two obtained distributions of variances (Figure S7C) showed that structurally variable domains exhibit higher epigenetic variance than conserved ones (one-sided Mann–Whitney U test, *p* = 1.60 × 10^−11^). Effect size was quantified using the median difference (0.003) and Cliff’s δ (0.28).

### 4.16. Structural Impact of Epigenetic Classes

To evaluate the contribution of each epigenetic class to population-level folding, we performed a series of in silico silencing experiments following the approach of ref. [13]. For each of the five epigenetic classes defined above, all domains belonging to that class were erased in each single-cell polymer by replacing their binding sites with inert beads. The modified polymers were summed to produce a mutated population-level polymer, from which a contact matrix was recomputed (Figure S8A). The structural impact of each mutation was quantified by computing the distance-corrected Pearson correlation (r′) between the resulting contact matrix and the bulk Hi-C map. The difference with respect to the correlation of the wild-type matrix (r′ = 0.60; Figure 3A) defines the correlation loss Δr′ for each class. Visual inspection of the resulting maps highlights distinct contact patterns that are lost depending on the erased class, many of which coincide with dense domains present in the bulk contact matrix (colored boxes in Figure S8A).

Since the five epigenetic classes differ in abundance (Figure S8B), we next considered whether the observed Δr′ values could be explained simply by the number of domains removed. To rigorously control for the effect of these differences, we generated a set of randomized mutations per each class. In each replicate, we first randomly reassigned each single-cell binding domain to one of the five epigenetic classes, independently of its true epigenetic profile. We then deleted all binding domains assigned to each of these randomized classes and recalculated the mutated polymer and its contact matrix, obtaining five Δr′ values for that iteration. Repeating this process across 30 replicates yielded a control distribution of 150 Δr′ values, reflecting the structural impact expected under randomized class assignments. The resulting control distribution of Δr′ values for size-matched mutations was then used to assess the significance of the structural impact of each epigenetic class (Figure S8C).

## Data Availability

This paper analyzes existing, publicly available data. Accession numbers for the datasets are listed in Section 4. Any additional information required to reanalyze the data reported in this paper is available from the lead contact upon request.

## Supplementary Materials

The following supporting information can be downloaded at: https://www.mdpi.com/article/10.3390/ijms27114803/s1. Reference [69] is cited in supplementary materials.
