# Supplementary material for: Physics-Based Modeling of Sparse Single-Cell Hi-C Uncovers Structural and Epigenetic Variability
Source: Int J Mol Sci. 2026 May 26;27(11):4803. doi: 10.3390/ijms27114803 (PMC13256433; doi:10.3390/ijms27114803)
Supplement: Supplementary file 1 [file ijms-27-04803-s001.zip › ijms-4190826-supplementary.pdf]

Supplementary Tables

Data input (%)	Median difference	Cliff's δ	P value BH adj
2	0,1557	0,4012	2.12e-02
10	0,1488	0,3791	2.51e-02
20	0,3224	0,5859	1.54e-03
30	0,4762	0,7717	1.11e-04
40	0,4956	0,7363	1.42e-04
50	0,3736	0,5931	1.54e-03
60	0,5307	0,779	1.11e-04
70	0,515	0,7993	1.11e-04
80	0,5066	0,5901	1.54e-03
90	0,5195	0,7427	1.42e-04

Table S1: Statistical significance of overlap distributions between downsampled and bulk Hi-C derived polymer models.

Statistical comparison between overlap distributions from models inferred from downsampled Hi-C data and corresponding random controls at each input percentage. For each condition, the median difference between real and control overlaps, Cliff's δ effect size, and Benjamini–Hochberg adjusted p-values from one-sided Mann–Whitney U tests are reported. All comparisons are statistically significant after multiple testing correction (adjusted $p < 0.05$) and show non-negligible effect sizes (Cliff's $\delta > 0.147$ [1]).

Color	Median difference	Cliff's δ	P value BH adj
1	0,1387	0,5624	2.87e-27
2	0,076	0,4231	3.87e-16
3	0,1503	0,4771	4.55e-20
4	-0,0256	-0,0999	5.17e-02
5	0,0661	0,418	7.00e-16
6	0,0916	0,6484	1.32e-35
7	0,0258	0,1581	2.67e-03
8	-0,0756	-0,3236	4.38e-10
9	0,0533	0,1031	5.02e-02

Table S2: Statistical analysis of overlap distributions for individual binding domain types.

Statistical comparison of overlap distributions between single cell polymers and random controls for each binding domain type. For each color, we report the median difference between real and control overlaps, Cliff's δ effect size, and Benjamini–Hochberg adjusted p-values from two-sided Mann–Whitney U tests. Results reveal heterogeneous behavior across domains, with several showing strong enrichment (e.g., colors 1 and 6), others moderate or weak effects, and some displaying depletion relative to random controls (e.g., color 8).

Supplementary Figures

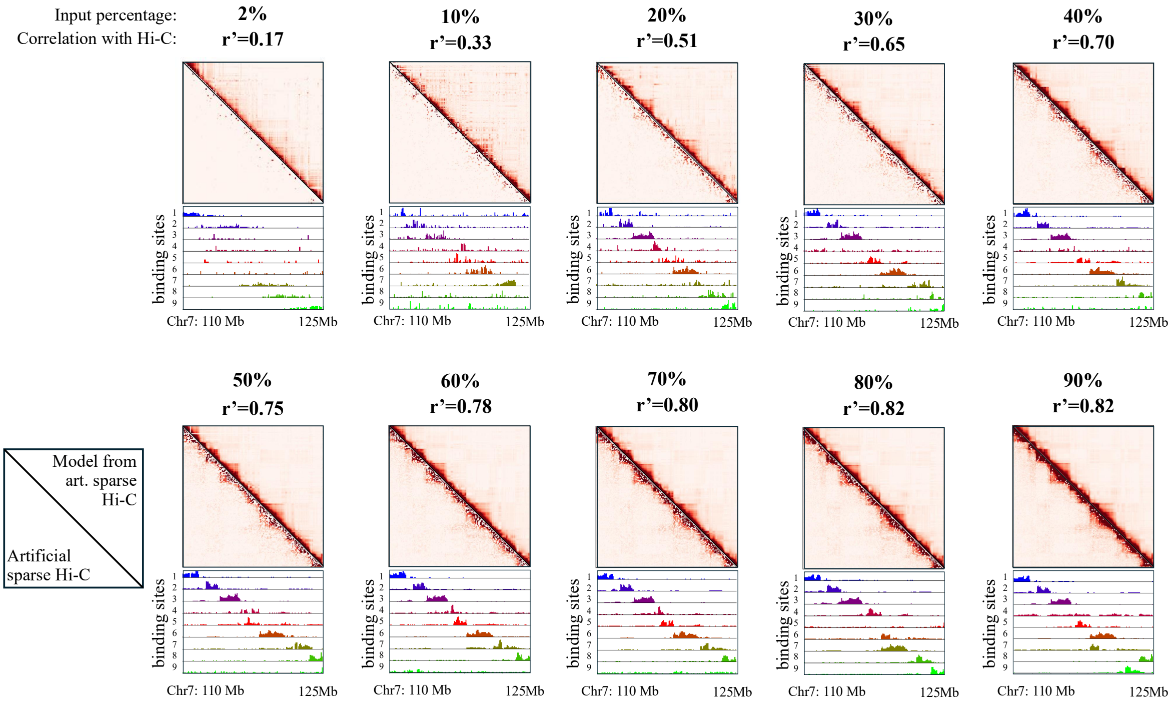

Figure S1: Models from downsampled Hi-C data.

Models derived from downsampled Hi-C matrices, produced by retaining increasing fractions of bulk Hi-C [2] contacts from 2% to 90%. For each downsampling level, the model-inferred contact matrix is shown above, and the corresponding SBS binding domain profile is shown below. r′ values with the original bulk Hi-C are indicated above each matrix. All contact maps are at 100 kb resolution for the HeLa-S3 cell locus (chr7:110–125 Mb).

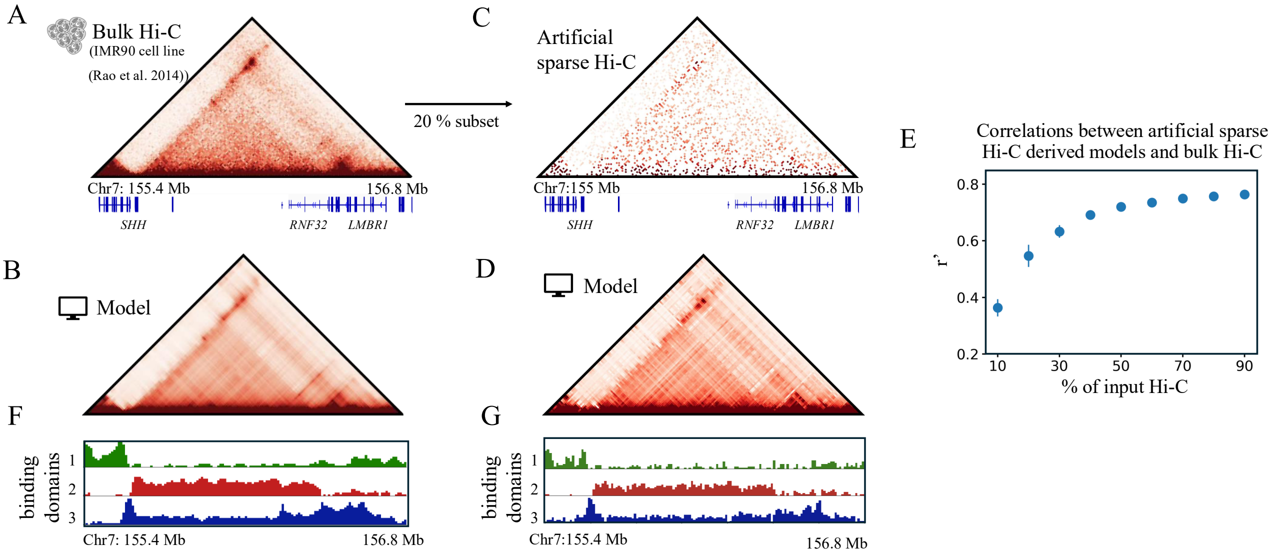

Figure S2: Model of the IMR90 cell locus from bulk and from downsampled Hi-C data.

(A) Bulk Hi-C [3] contact matrix of a 1.5Mb-wide locus (chr7:155.4–156.8 Mb) at 10kb resolution in IMR90 cells.

(B) Contact matrix of the polymer model inferred from bulk Hi-C of the IMR90 cell locus has a distance-corrected correlation with bulk Hi-C of r′ =0.77.

(C) Example of artificial sparse Hi-C matrix of the locus obtained by randomly downsampling the bulk Hi-C matrix to 20% of its original entries.

(D) Contact matrix of the polymer model inferred from the artificial sparse Hi-C matrix in panel C. Its distance-corrected correlation with the bulk Hi-C matrix (**panel A**) is r′ =60.

(E) Distance-corrected Pearson correlation, r′, between the model contact matrices inferred from artificial sparse Hi-C at different downsampling percentages and bulk Hi-C matrix. Each point is averaged over 20 independent random realizations; error bars indicate standard deviation.

(F) Polymer model binding domains inferred from bulk Hi-C.

(G) Polymer model binding domains inferred from the artificial sparse Hi-C in panel C.

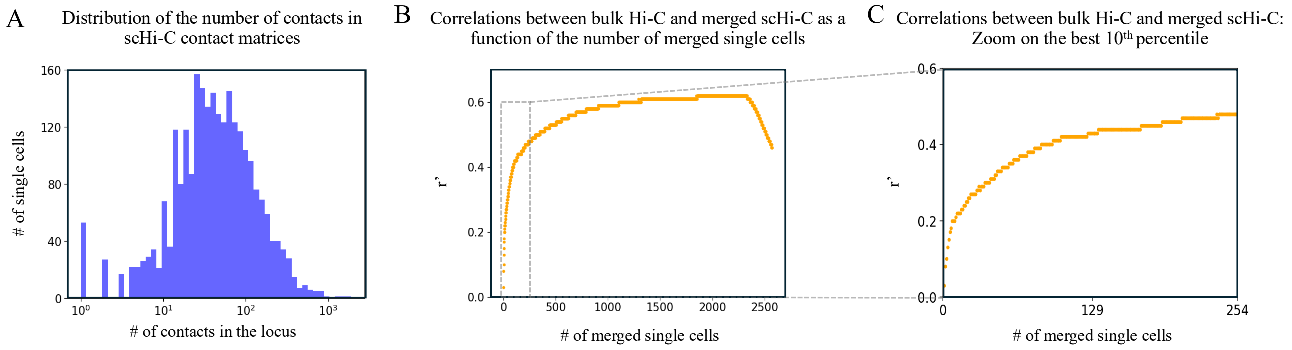

Figure S3: Characterization of the analyzed single cell Hi-C data.

(A) Distribution of the number of scHi-C contacts in-cis within the HeLa-S3 cell locus (chr7:110–125 Mb) across the 2,574 single cells from ref. [4] with at least one contact in the locus. The top 5% of single cells (129 in total) were selected for polymer modeling, representing a trade-off between locus-specific coverage and population size.

(B) Distance-corrected Pearson correlation (r′) between the bulk Hi-C matrix and the merged contact matrix from the top *n* single cell Hi-C matrices, ordered by contact number in the selected locus. The correlation reaches ~70% of its maximum already with the top 5% (129 cells), supporting this selection as sufficient to capture population-level structural features within the locus.

(C) Zoom of panel **(B)** focusing on the top 10% of single cells (254 cells) ranked by contact number within the locus. The distance-corrected Pearson correlation (r′) between bulk Hi-C and merged single cell matrices increases with the number of included cells, reaching r′ ≈ 0.48 with 254 cells. Notably, using only the top 5% of cells (129 cells) already yields r′ ≈ 0.43, corresponding to ~90% of the value observed for the 10%, supporting this threshold as an effective trade-off between data coverage and population size.

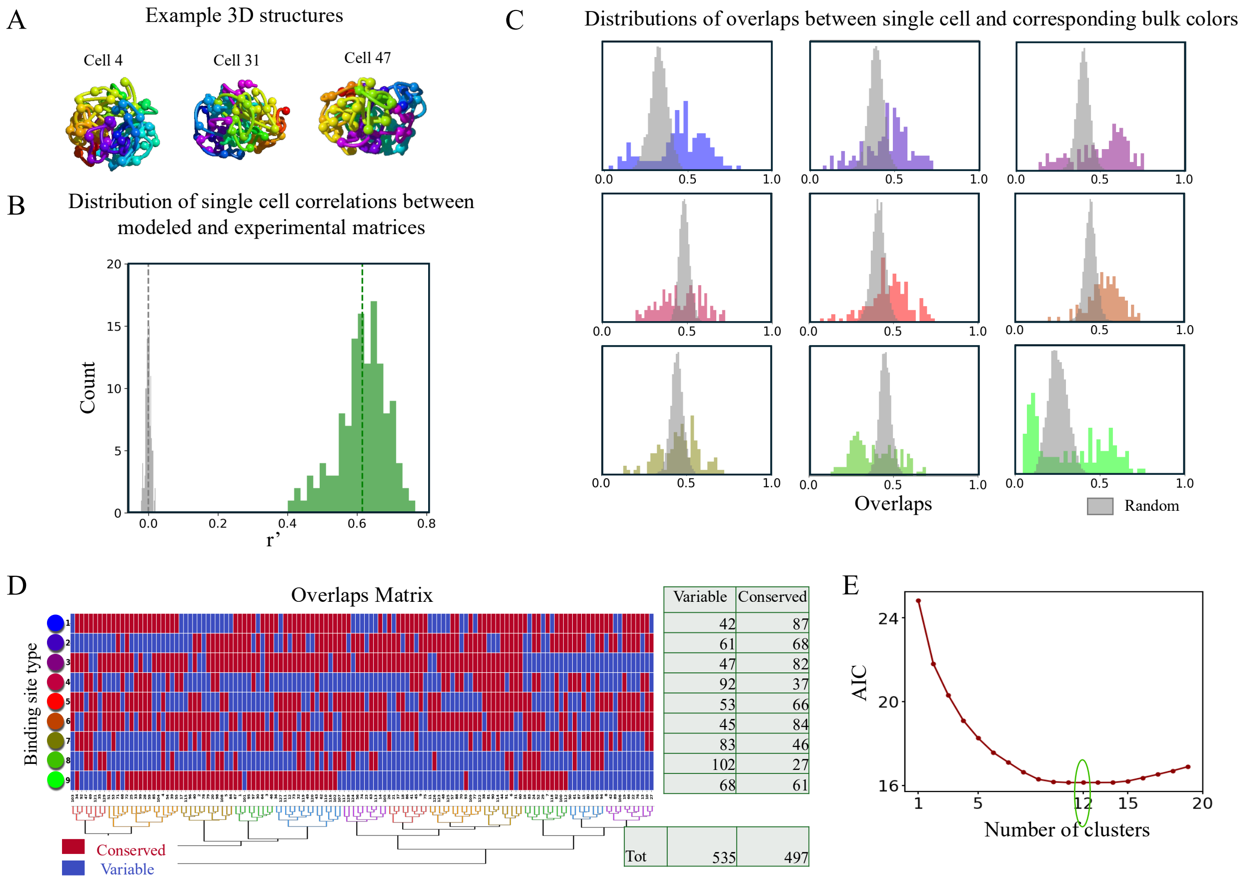

FIGURE S4: Validation and structural variability of single cell models.

(A) Additional examples of 3D conformations for the three single cell polymer models of **Fig. 2.** See **Fig. 2B** for 3D color scheme**.**

(B) Distribution of distance-corrected Pearson correlation (r′) between modeled single cell contact matrices and the corresponding experimental single-cell Hi-C matrices (green). Gray distribution represents a control obtained by independently shuffling contact values within each diagonal of the inferred matrices, preserving distance-dependent contact decay while disrupting genomic organization. Our method consistently outperforms the control across cells. The per-cell difference Δr′ is strictly positive (Wilcoxon signed-rank test, $p = 6.51 \times 10^{-23}$), with a median difference of 0.62 and Cliff's $\delta = 1$, indicating complete separation between conditions.

(C) Overlap distributions between each single cell polymer and the bulk Hi-C derived SBS model, for each of the nine binding domain types (colors). Gray distributions are from random controls built by 50 single cell polymers bootstrappings. Statistical significance was assessed using two-sided Mann–Whitney U tests with Benjamini–Hochberg correction for multiple comparisons. Effect sizes, quantified by median differences and Cliff's δ, reveal heterogeneous behavior across domains, with several colors showing strong enrichment and others weaker or even depleted overlap relative to random controls (**Table S2**).

(D) Domain conservation across individual single cell polymers. The heatmap shows, for each cell (column) and each of the nine binding domain types (rows), whether a given domain is *conserved* (red) or *variable*

(blue) compared to the bulk. A domain is considered conserved when its overlap with the bulk exceeds the 90th percentile of a control distribution generated via bootstrapping. The dendrogram below reflects hierarchical clustering of single cell polymers based on their domain conservation profiles. The right panel summarizes, for each cluster, the number of conserved and variable domains.

(E) Selection of the number of structural clusters based on the Akaike Information Criterion (AIC, [5]), identifying 12 as the optimal number of clusters (green circle).

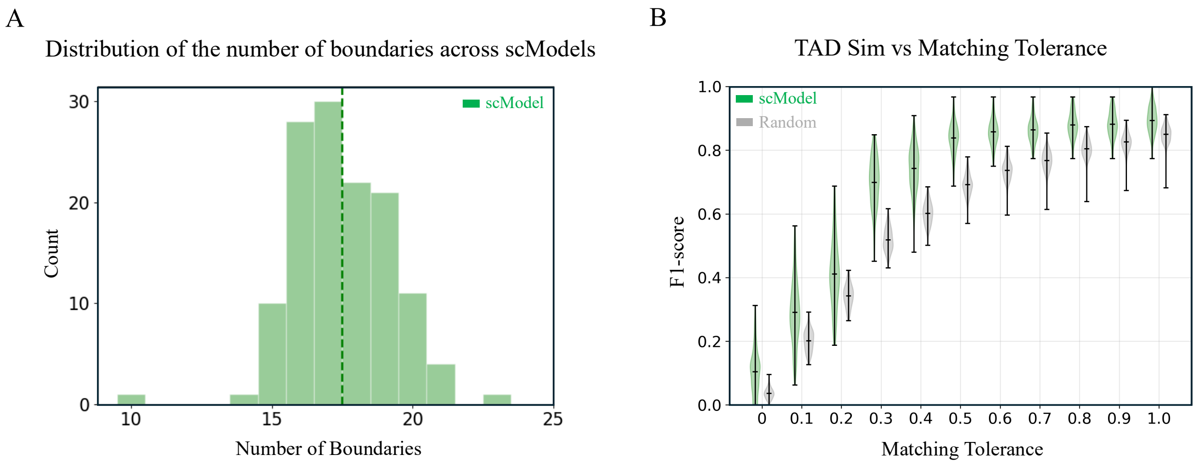

Figure S5: Single cell TAD boundaries variability and robustness to matching tolerance.

(A) Distribution of the number of TAD boundaries identified per cell across all 129 single cell polymer models. Dashed green line indicates the mean value of the distribution. On average, single cell polymer models produced 17.5 ± 1.8 boundaries.

(B) Dependence of the TAD similarity F_1 score [6] on the matching tolerance parameter. Model-derived boundaries (green) consistently outperform random controls (gray) across all tested values. The curve exhibits a clear elbow around 0.3 Mb, indicating an optimal trade-off between sensitivity and specificity. Nearby values (e.g. 0.4 Mb) yield similar performance, supporting the robustness of the chosen threshold.

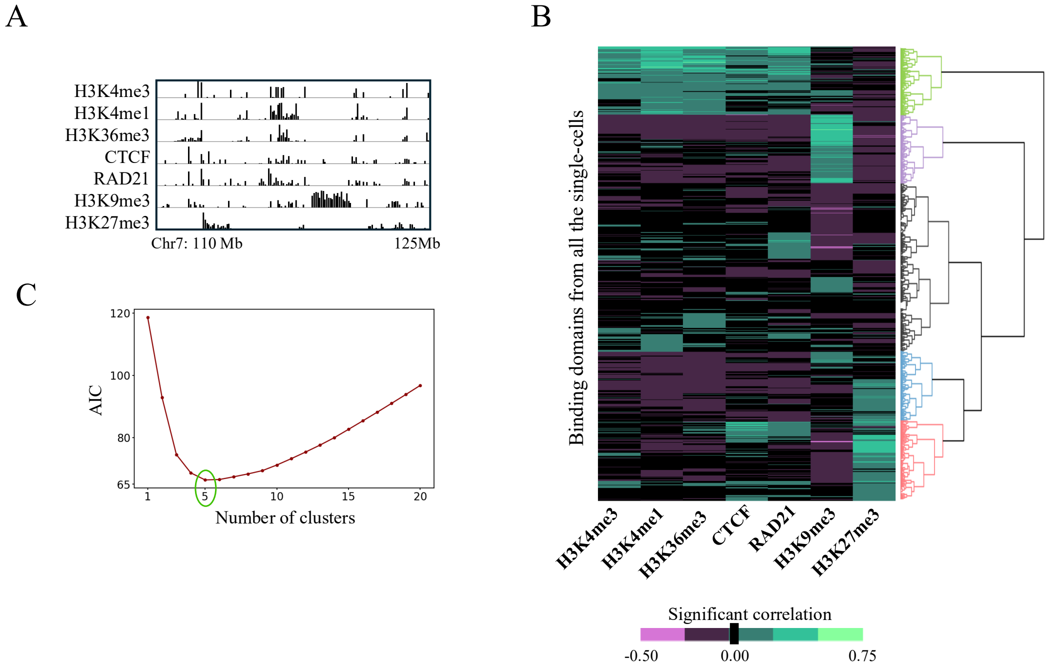

FIGURE S6: Identification of single cell epigenetic classes.

(A) Genomic profiles of seven bulk epigenetic signals (z-score normalized) across the HeLa –S3 cell (locus chr7:110–125 Mb), binned at 100 kb resolution. Signals were obtained from ENCODE [7] (see **Methods**).

(B) Pearson correlations between the seven bulk signals and the binding domains from the 129 single cell polymers. Each row represents one domain, each column one signal. Black entries indicate non-significant correlations (see **Methods**).

(C) Akaike Information Criterion (AIC, [5]) for increasing numbers of clusters, supporting the selection of the five epigenetic classes used in **Figure 5**.

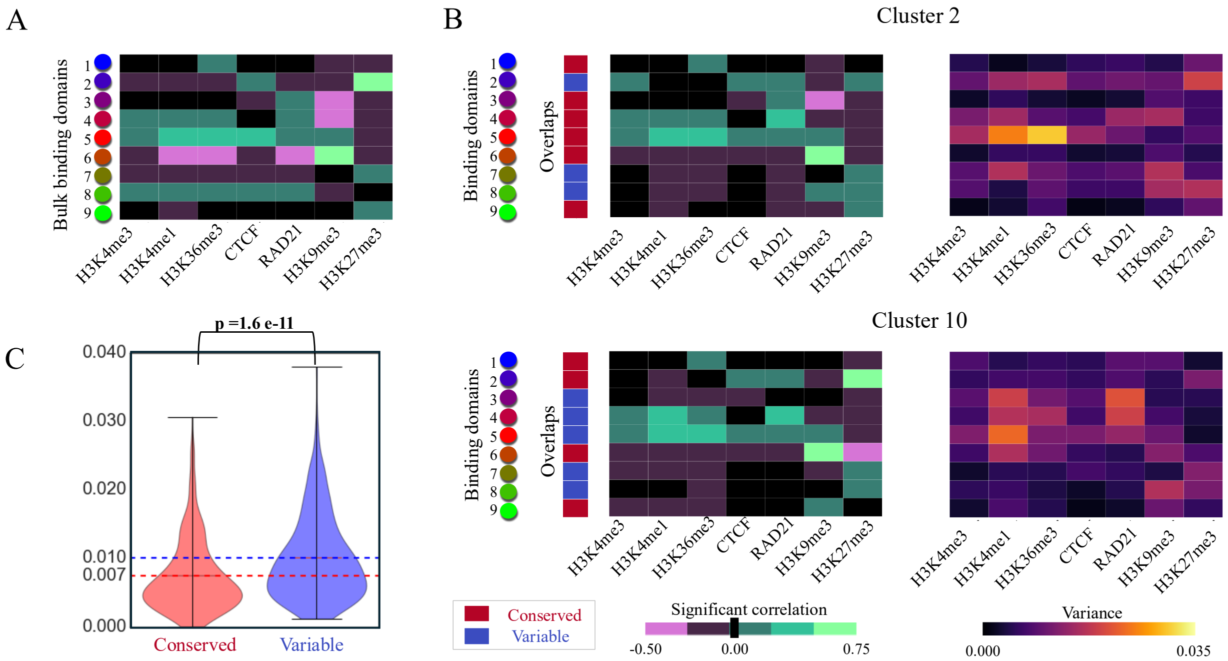

FIGURE S7: Epigenetic profiles of structural clusters.

(A) Epigenetic profiles of the binding domains of the bulk Hi-C derived SBS polymer model.

(B) Epigenetic profiles of the merged binding domains (**Figure 2G**) from structural Clusters 2 (top left) and 10 (bottom left). The heatmaps on the right shows the variance of the epigenetic signature across across all single cells in the cluster, for cluster 2 (top right) and 10 (bottom right) respectively. The colored box on the left indicates whether each domain is classified as conserved (red) or variable (blue), based on its overlap with the bulk polymer (see **Figure 2E**).

(C) Distribution of intra-cluster epigenetic variances from all the clusters, grouped according to the structural label of each domain (conserved or variable). Structurally variable binding domains exhibit higher epigenetic variability compared to conserved domains (one-sided Mann–Whitney U test, $p = 1.6\times10^{-11}$; median difference = 0.003; Cliff's $\delta = 0.28$). Dashed lines indicate the average variance within each group.

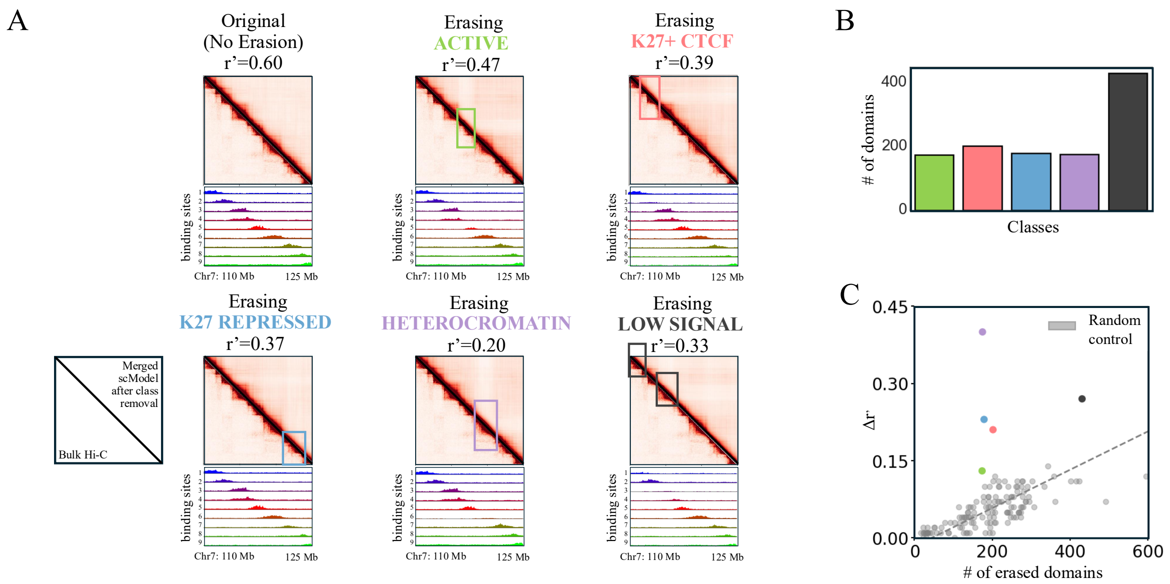

FIGURE S8: Structural contribution of each epigenetic class.

(A) Population-level contact matrices (CM, top panels) and associated polymer binding domains (bottom panels) obtained after in silico erasure of all binding domains assigned to one epigenetic class at a time. The distance-corrected Pearson correlation (r′) with the bulk Hi-C matrix is reported for each condition.

(B) Number of single cell binding domains in each epigenetic class.

(C) Scatter plot showing the loss in correlation (Δr′) with the bulk Hi-C matrix after *in silico* erasure of each epigenetic class (colored dots), against the number of erased domains. Gray dots represent control values obtained from randomized mutations (see **Methods**). The dashed line shows a linear fit over the random control points, indicating the expected correlation loss as a function of the number of erased domains. All five observed epigenetic classes lie above the random expectation, indicating that their removal causes a greater structural disruption than expected based only on their number.

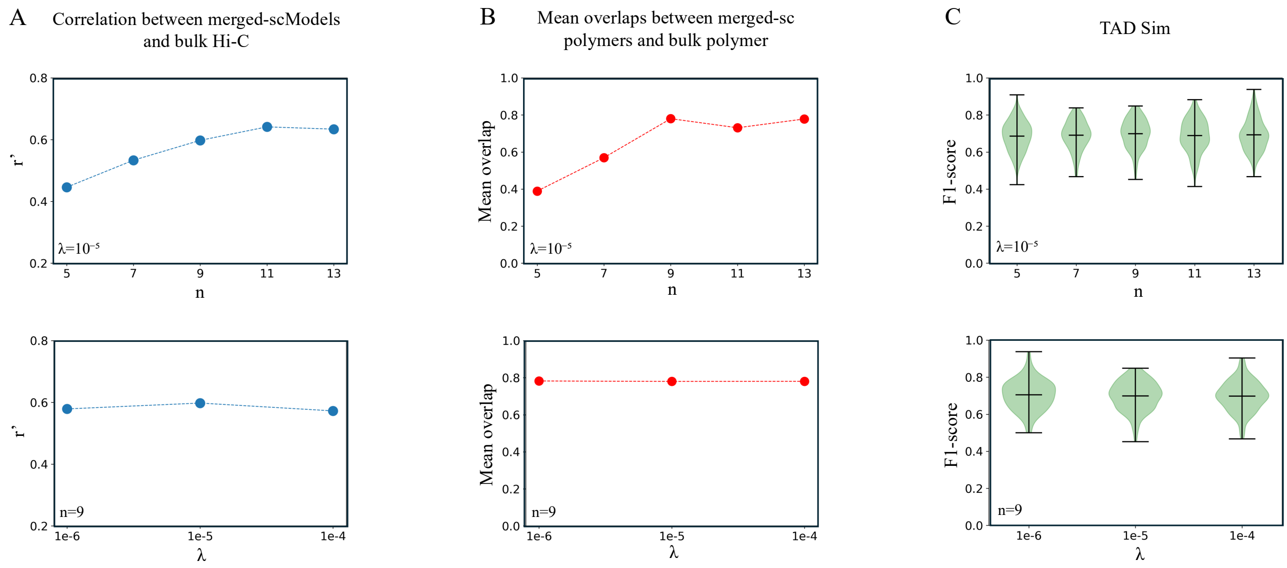

FIGURE S9: Robustness of main results to the model parameters.

(A) Distance-corrected Pearson correlation (r′) between bulk Hi-C and merged single cell model contact matrices obtained by varying the number of PRISMR colors, n, at fixed $\lambda = 10^{-5}$ (top), and by varying λ at fixed n = 9 (bottom).

(B) Mean overlap between merged single cell polymers and the bulk-derived polymer model across the same parameter settings.

(C) Distribution of TAD similarity F_1 scores between single cell model-derived boundaries and bulk boundaries across the same parameter settings. Overall, the three readouts remain stable around the parameters used in the main analysis (n = 9, $\lambda = 10^{-5}$), supporting the robustness of the main conclusions to variations in PRISMR parameters.

Supplementary References

1. Wan, Z.; Xia, X.; Lo, D.; Murphy, G.C. How Does Machine Learning Change Software Development Practices? *IEEE Transactions on Software Engineering* **2020**, 1–1, doi:10.1109/TSE.2019.2937083.

2. Akgol Oksuz, B.; Yang, L.; Abraham, S.; Venev, S. V.; Krietenstein, N.; Parsi, K.M.; Ozadam, H.; Oomen, M.E.; Nand, A.; Mao, H.; et al. Systematic Evaluation of Chromosome Conformation Capture Assays. *Nat. Methods* **2021**, *18*, 1046–1055, doi:10.1038/s41592-021-01248-7.

3. Rao, S.S.P.; Huntley, M.H.; Durand, N.C.; Stamenova, E.K.; Bochkov, I.D.; Robinson, J.T.; Sanborn, A.L.; Machol, I.; Omer, A.D.; Lander, E.S.; et al. A 3D Map of the Human Genome at Kilobase Resolution Reveals Principles of Chromatin Looping. *Cell* **2014**, *159*, doi:10.1016/j.cell.2014.11.021.

4. Ramani, V.; Deng, X.; Qiu, R.; Gunderson, K.L.; Steemers, F.J.; Disteche, C.M.; Noble, W.S.; Duan, Z.; Shendure, J. Massively Multiplex Single-Cell Hi-C. *Nat. Methods* **2017**, *14*, 263–266, doi:10.1038/nmeth.4155.

5. Akaike, H. A New Look at the Statistical Model Identification. *IEEE Trans. Automat. Contr.* **1974**, *19*, 716–723, doi:10.1109/TAC.1974.1100705.

6. Murtaza, G.; Butaney, B.; Wagner, J.; Singh, R. ScGrapHiC: Deep Learning-Based Graph Deconvolution for Hi-C Using Single Cell Gene Expression. *Bioinformatics* **2024**, *40*, i490–i500, doi:10.1093/bioinformatics/btae223.

7. Dunham, I.; Kundaje, A.; Aldred, S.F.; Collins, P.J.; Davis, C.A.; Doyle, F.; Epstein, C.B.; Frietze, S.; Harrow, J.; Kaul, R.; et al. An Integrated Encyclopedia of DNA Elements in the Human Genome. *Nature* **2012**, *489*, doi:10.1038/nature11247.
